# Bioactivity-Guided Isolation of Cytoprotective Metabolites from Two *Verbascum* Species Reveals Differential Modulation of Cigarette Smoke-Induced Inflammation and Oxidative Stress

**DOI:** 10.3390/molecules31213818

**Published:** 2026-10-22

**Authors:** Gulin Renda, Nurdan Yazici, Rengin Reis, Elif Nur Barut, Burak Barut, Icim Gokkaya, Elif Oz, Manar M. Amin, Yeter Yesil Canturk

**Affiliations:** 1 Department of Pharmacognosy, Faculty of Pharmacy, Karadeniz Technical University, Trabzon 61080, Türkiye; grenda@ktu.edu.tr (G.R.); nurdanyazici@ktu.edu.tr (N.Y.); 2 Trabzon Teknokent, Farmakodental Health Products, Trabzon 61080, Türkiye; 3 Department of Toxicology, Faculty of Pharmacy, Acibadem Mehmet Ali Aydinlar University, Istanbul 34752, Türkiye; elif.oz@acibadem.edu.tr; 4 Department of Pharmacology, Faculty of Pharmacy, Karadeniz Technical University, Trabzon 61080, Türkiye; elifgazioglu@ktu.edu.tr; 5 Department of Biochemistry, Faculty of Pharmacy, Karadeniz Technical University, Trabzon 61080, Türkiye; burakbarut@ktu.edu.tr; 6 Department of Pharmaceutical Botany, Faculty of Pharmacy, Karadeniz Technical University, Trabzon 61080, Türkiye; icimgokkaya@ktu.edu.tr; 7 Institute of Health Sciences, Ankara University, Ankara 06290, Türkiye; 8 Institute of Graduate Studies in Health Sciences, Istanbul University, Istanbul 34126, Türkiye; 9 Department of Pharmacology, Faculty of Pharmacy, University of Khartoum, Khartoum 11111, Sudan; manarmustafaamin97@gmail.com; 10 Department of Pharmacology, Graduate School of Health Sciences, Karadeniz Technical University, Trabzon 61080, Türkiye; 11 Department of Pharmaceutical Botany, Faculty of Pharmacy, Istanbul University, Istanbul 34126, Türkiye

**Keywords:** activity guided isolation, cigarette smoke extract, BEAS-2B, inflammation

## Abstract

*Verbascum* species are traditionally used for respiratory disorders and are rich sources of iridoid glycosides, phenylethanoid glycosides, and triterpenoid saponins. However, *V. kurdicum* and *V. longipedicellatum* have not previously been investigated phytochemically or biologically. This study aimed to identify their bioactive constituents and characterize their protective effects against cigarette smoke extract (CSE)-induced bronchial epithelial injury. Bioactivity-guided fractionation of 50% methanol extracts yielded twelve compounds identified by NMR. Cytoprotective potential was evaluated in BEAS-2B cells, and selected compounds were investigated for effects on IL-6, IL-8, HO-1, TLR4 expression and downstream effectors (MyD88 and NF-κB), GSH, 8-OHdG levels, and SOD activity. Ajugol exhibited the strongest cytoprotective activity and significantly restored viability following CSE exposure. Ajugol, aucubin, and 1-*O*-caffeoyl-β-D-glucopyranose were selected for mechanistic evaluation. Aucubin and 1-*O*-caffeoyl-β-D-glucopyranose significantly reduced CSE-induced IL-6 release, while all three suppressed IL-8, with ajugol producing the greatest effect. Ajugol also attenuated TLR4 upregulation and reduced 8-OHdG levels and the CSE-induced increase in SOD activity, whereas MyD88, total NF-κB, p-NF-κB^Ser536^, and GSH were not significantly improved. These findings provide the first phytochemical and biological characterization of both species and identify ajugol as a promising lead against CSE-induced bronchial epithelial damage.

## 1. Introduction

Cigarette smoke (CS) is a complex mixture of chemicals capable of inducing oxidative stress, inflammatory signaling, and cellular injury in the respiratory epithelium. Bronchial epithelial cells constitute the primary cellular barrier against inhaled toxicants and contribute actively to pulmonary immune responses through the release of cytokines and chemokines [1,2]. CS is recognized as the most significant risk factor in the development of chronic obstructive pulmonary disease (COPD) and other chronic lung diseases. Moreover, according to the World Health Organization, COPD is among the leading causes of death worldwide [3]. Exposure to CS causes direct damage to the bronchial epithelial cells, which serves as the primary barrier and initiator of immune responses in the lungs. In the inflammatory response triggered by CS, specific molecular pathways involving cytokines such as interleukin-6 (IL-6) and interleukin-8 (IL-8), alongside receptor complexes like Toll-like receptor 4 (TLR4) and nuclear factor-kappa B (NF-κB), play crucial roles in maintaining and amplifying chronic inflammation [4,5]. Concurrently, persistent oxidative stress overpowers endogenous protective mechanisms, causing direct genotoxic damage. Given the irreversible and progressive nature of airway remodeling in COPD, discovering safe and effective protective compounds that target these specific epithelial damage pathways is of utmost importance [2,6]. To achieve this, researchers have increasingly transitioned toward natural products, which offer structurally diverse, bioactive chemical scaffolds as potential leads for therapeutic intervention.

*Verbascum* L., the largest genus of the family Scrophulariaceae, comprises approximately 360 species worldwide. Although members of the genus are distributed across Europe, Asia, and Northeast Africa, their distribution is markedly uneven, with the principal center of diversity occurring in Turkey and Iran. Turkey constitutes a major diversity hotspot, harboring 248 species and 129 hybrids [7]. Various species of this genus, commonly known as mullein, represent a highly rational source for screening bioactive compounds due to their extensive ethnopharmacological relevance and established phytochemistry [8,9]. Traditionally, *Verbascum* species have been utilized for centuries in global and Turkish folk medicine to treat respiratory disorders such as asthma, bronchitis, dry cough, and tuberculosis [10,11,12,13]. Indeed, the flowers of *Verbascum thapsus* L., *Verbascum densiflorum* Bertol, and *Verbascum phlomoides* L. are officially recognized by the European Medicines Agency (EMA) as traditional herbal medicinal products for the symptomatic treatment of dry coughs and upper respiratory tract irritation [14]. This widespread traditional use is strongly supported by their characteristic chemical composition, which is highly rich in oleanane-type triterpenoid saponins, iridoid glycosides, phenylethanoid glycosides, and flavonoids. Modern pharmacological investigations have validated these applications, showing that *Verbascum* extracts and their isolated secondary metabolites exhibit potent anti-inflammatory, antioxidant, immunomodulatory, and wound-healing activities [15,16]. Numerous in vitro and in vivo studies have demonstrated that these characteristic metabolites significantly suppress key inflammatory mediators and pathways, including IL-6, IL-8, iNOS, and NF-κB [17,18,19,20]. Consequently, the genus *Verbascum* is considered a highly promising natural resource containing bioactive compounds suited for treating inflammatory respiratory diseases.

Despite the rich ethnopharmacological history and remarkable chemical diversity of the genus, the potential of *Verbascum* species to protect bronchial epithelial cells against CS-induced injury remains poorly explored. Prior research on the pulmonary activities of *Verbascum* has predominantly focused on anticancer screening against lung cancer lines or evaluated the effects of individual compounds, such as verbascoside, in experimental models of asthma and acute lung injury [6,21,22,23]. However, prior to this study, there were no reports evaluating the protective or anti-inflammatory potential of *Verbascum* extracts or their isolated iridoids, saponins, and phenylethanoids in models of CS-induced lung injury. Among the diverse *Verbascum* taxa occurring in Türkiye, *Verbascum longipedicellatum* Hub.-Mor. and *Verbascum kurdicum* Hub.-Mor. remained entirely unexplored from both phytochemical and biological perspectives. Geographically and taxonomically, both taxa belong to Group I in the Flora of Turkey but exhibit distinct morphological features and distribution patterns. *V. longipedicellatum* is an endemic species restricted to high-altitude subalpine meadows (1650–2000 m) on rocky slopes, whereas *V. kurdicum* is an Iran-Turan element whose type specimen originates from Türkiye, typically growing in limestone rocky slopes and Quercus scrublands at lower elevations [24]. Given their complete lack of previous scientific characterization, these two species represent excellent candidates for bioactivity screening. Moreover, no bioactivity-guided investigation has yet linked the chemical constituents of these species with protection against CS-induced bronchial epithelial injury in vitro.

Therefore, the present study aimed to investigate the cytoprotective, anti-inflammatory, and protective potential of *V. kurdicum* and *V. longipedicellatum* against oxidative damage using a systematic and integrated bioactivity-guided fractionation strategy. The experimental funnel design began with extracting the aerial parts of both species using 50% methanol, followed by liquid–liquid partitioning with *n*-hexane and *n*-butanol. The resulting fractions were initially screened for their cytoprotective capacity and ability to suppress IL-6 secretion in an in vitro model of cigarette smoke extract (CSE)-induced injury in human BEAS-2B bronchial epithelial cells. With this in vitro model, it was aimed to provide a controlled and reproducible approach for investigating early epithelial responses to CS and for the preliminary screening of compounds that may modulate CSE-induced cellular injury and inflammatory signaling. The highly active *n*-butanol fractions (VK1A and VL1E) were then prioritized for chromatographic purification. This purification led to the isolation and spectroscopic structure elucidation (via 1D/2D NMR and MS) of several active constituents, including iridoid glycosides, phenylethanoid glycosides, saponins, and nucleosides. These isolated compounds—specifically ajugol, aucubin, and 1-O-caffeoyl-β-D-glucopyranose—were subsequently screened in the CSE-induced BEAS-2B cell injury model. To establish their molecular mechanisms, the protective effects of these lead compounds were characterized by analyzing key biomarkers of lung inflammation and oxidative stress, including IL-6, IL-8, HO-1, SOD, GSH, 8-OHdG, and the TLR4-associated signaling components. This integrated strategy was designed to identify the constituents responsible for the protective effects of these previously unexplored *Verbascum* species and to characterize their differential biological profiles against CS-induced epithelial damage in vitro.

## 2. Results

### 2.1. Bioactivity-Guided Selection of Active Fractions from V. kurdicum and V. longipedicellatum

As the initial step in the bioactivity-guided fractionation strategy, the crude extracts and fractions obtained from *V. kurdicum* and *V. longipedicellatum* were evaluated for their effects on BEAS-2B cell viability. Cells were exposed to five concentrations of each sample for 24 h, and concentrations maintaining ≥70% viability relative to the vehicle control were considered non-cytotoxic and selected for subsequent cytoprotective screening. The extracts and fractions displayed concentration-dependent differences in their cytotoxicity profiles (Supplementary Figures S1 and S2). Based on these results, the highest non-cytotoxic concentration of each sample was selected for subsequent cytoprotective screening (Supplementary Table S1). Among the polyamide fractions subsequently selected as the most promising candidates, VK1A and VL1E were tested at 0.01 and 0.1 mg/mL, respectively.

**Figure 1:**
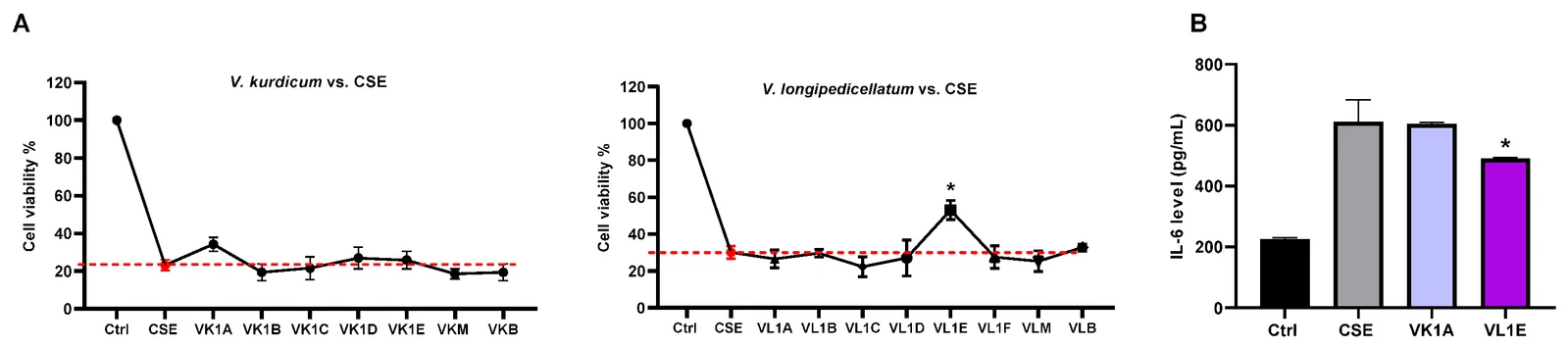
Bioactivity-guided selection of cytoprotective fractions from *V. kurdicum* and *V. longipedicellatum* against CSE-induced BEAS-2B cell injury. (**A**) Cytoprotective effects of *V. kurdicum* and *V. longipedicellatum* extracts and fractions. (**B**) Effects of the selected fractions VK1A and VL1E on CSE-induced IL-6 release. Cells were pretreated with the extracts or fractions for 20 h and subsequently exposed to 20% CSE (*v*/*v*) for 4 h. Concentrations used for cytoprotective screening are provided in Supplementary Table S1. Statistical significance versus the CSE-treated group: * *p* < 0.05. CSE, cigarette smoke extract (*n* = 3).

The non-cytotoxic concentrations listed in Supplementary Table S1 were then evaluated for their ability to protect BEAS-2B cells against CSE-induced injury. Exposure to 20% CSE for 4 h markedly reduced cell viability, confirming the establishment of cellular injury under the experimental conditions. Among the *V. kurdicum* samples, VK1A exhibited the highest cytoprotective effect (Figure 1A), whereas VL1E significantly increased cell viability compared with the CSE-treated group (*p* < 0.05) and was the most prominent fraction obtained from *V. longipedicellatum* (Figure 1A). These findings identified VK1A and VL1E as the principal cytoprotective fractions and provided the first biological criterion for their selection in the bioactivity-guided isolation process. The remaining fractions, including VK1E, showed less favorable cytoprotective profiles under the screening conditions and were therefore not pursued further for compound isolation. To further discriminate the biological profiles of these two fractions, their effects on CSE-induced IL-6 release were evaluated. Pretreatment with VK1A (0.01 mg/mL) significantly reduced IL-6 release compared with cells exposed to CSE alone (*p* < 0.05), whereas a statistically significant inhibitory effect was not observed for VL1E (Figure 1B). Thus, VK1A combined cytoprotective activity with significant suppression of the CSE-induced inflammatory response, while VL1E was selected primarily on the basis of its prominent cytoprotective activity.

### 2.2. Bioactivity-Guided Isolation and Structural Identification of Compounds

**Figure 2:**
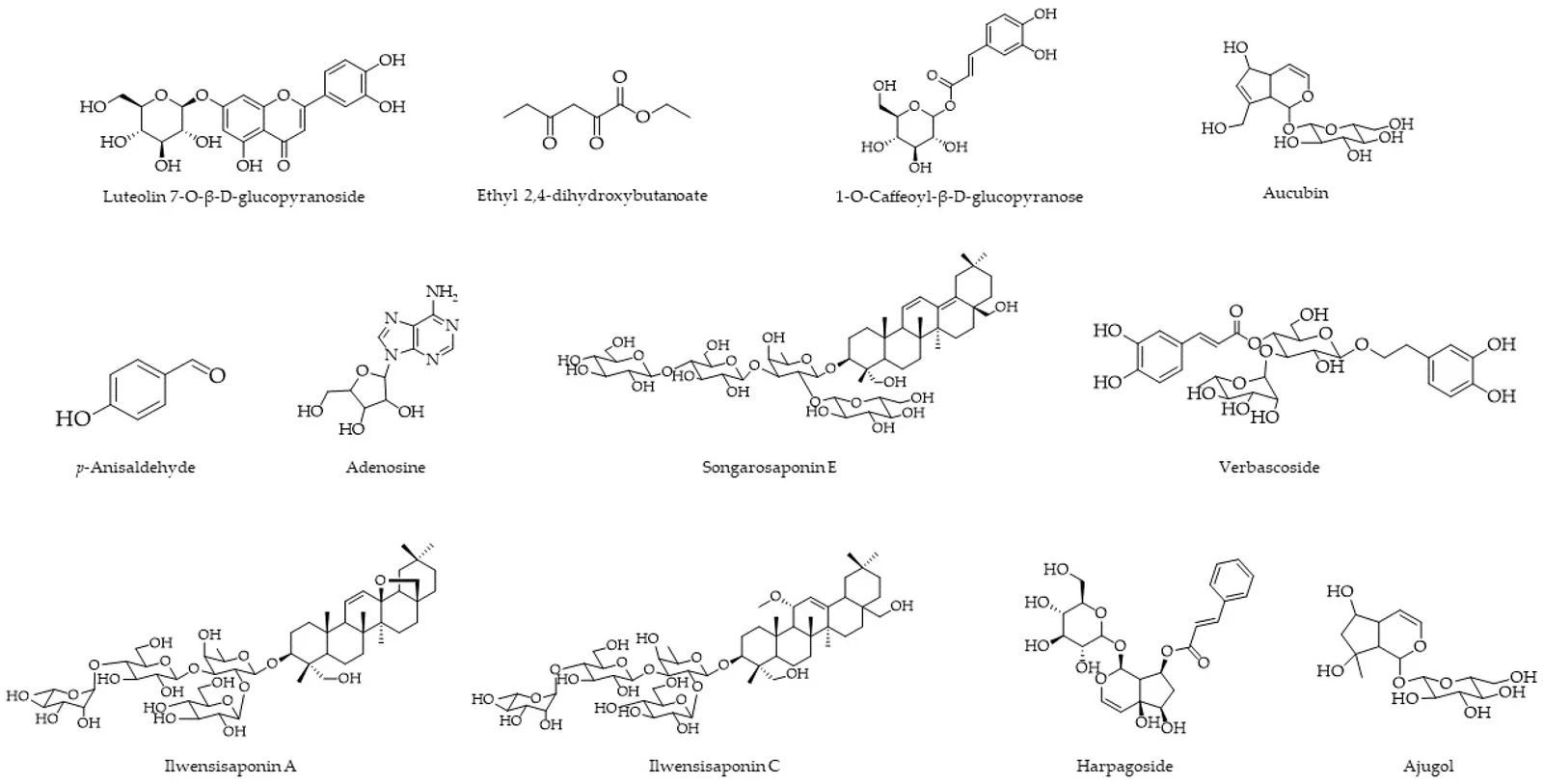
Chemical structures of compounds isolated from the bioactive fractions of *V. longipedicellatum* and *V. kurdicum*.

Bioactivity-guided chromatographic fractionation of VL1E and VK1A, the active fractions obtained from *V. longipedicellatum* and *V. kurdicum*, respectively, afforded a total of 12 compounds. The structures of the isolated compounds were identified by analysis of their 1D and 2D NMR data and comparison with previously reported spectroscopic data. The structures of all identified compounds are presented in Figure 2, while the corresponding spectroscopic data and spectra are provided in the Supplementary Material (Figures S3–S14). HPLC-DAD chromatographic purity assessment was additionally performed for four isolated compounds for which sufficient material was available, and the corresponding analytical conditions, purity values, and chromatograms are provided in the Supplementary Materials (Table S2 and Figure S15).

Purification of VL1E yielded four compounds: luteolin 7-O-β-D-glucopyranoside (LO-1, 24.2 mg), ethyl 2,4-dihydroxybutanoate (LO-2, 1.0 mg), 1-O-caffeoyl-β-D-glucopyranose (LO-4, 4.7 mg), and *p*-anisaldehyde (LO-5, 7.4 mg) (Figure 2). Their identities were established by comparison of the obtained spectroscopic data with previously reported data for luteolin 7-O-β-D-glucopyranoside [25], ethyl 2,4-dihydroxybutanoate [26], 1-*O*-caffeoyl-β-D-glucopyranose [27,28], and *p*-anisaldehyde [29], respectively. The assignments reported for LO-1, LO-2, LO-4, and LO-5 are supported by the corresponding NMR data in the study report.

Chromatographic purification of VK1A afforded eight identified compounds: adenosine (KUR-2, 2.4 mg), ajugol (KUR-3, 46.0 mg), aucubin (KUR-4, 3.9 mg), ilwensisaponin C (KUR-5, 7.8 mg), songarosaponin E (KUR-6, 4.1 mg), ilwensisaponin A (KUR-7, 4.0 mg), verbascoside (KUR-8, 2.7 mg), and harpagoside (KUR-9, 3.3 mg) (Figure 2). The spectroscopic assignments were consistent with previously reported data for adenosine [30], ajugol [31], aucubin [32], ilwensisaponin C [31], songarosaponin E [33], ilwensisaponin A [31], verbascoside [34], and harpagoside [34].

### 2.3. Cytotoxicity and Cytoprotective Effects of the Isolated Compounds

The 12 compounds isolated from the active fractions of *V. kurdicum* and *V. longipedicellatum* were subsequently evaluated for cytotoxicity and cytoprotective activity in BEAS-2B cells. Cytotoxicity was first assessed after 24 h of exposure to establish appropriate concentrations for the subsequent CSE challenge experiments (Supplementary Figure S15). Among the tested compounds, ilwensisaponin A (KUR7) reduced cell viability below the predefined 70% threshold at 10–100 µM; therefore, 0.1 µM was selected as a non-cytotoxic concentration for subsequent experiments. In addition, precipitation of songarosaponin E (KUR6) and harpagoside (KUR9) was observed in the culture medium at higher concentrations, and non-precipitating concentrations of 0.01 and 1 µM, respectively, were consequently used. The concentrations selected for cytoprotective screening of all isolated compounds are summarized in Supplementary Table S3.

**Figure 3:**
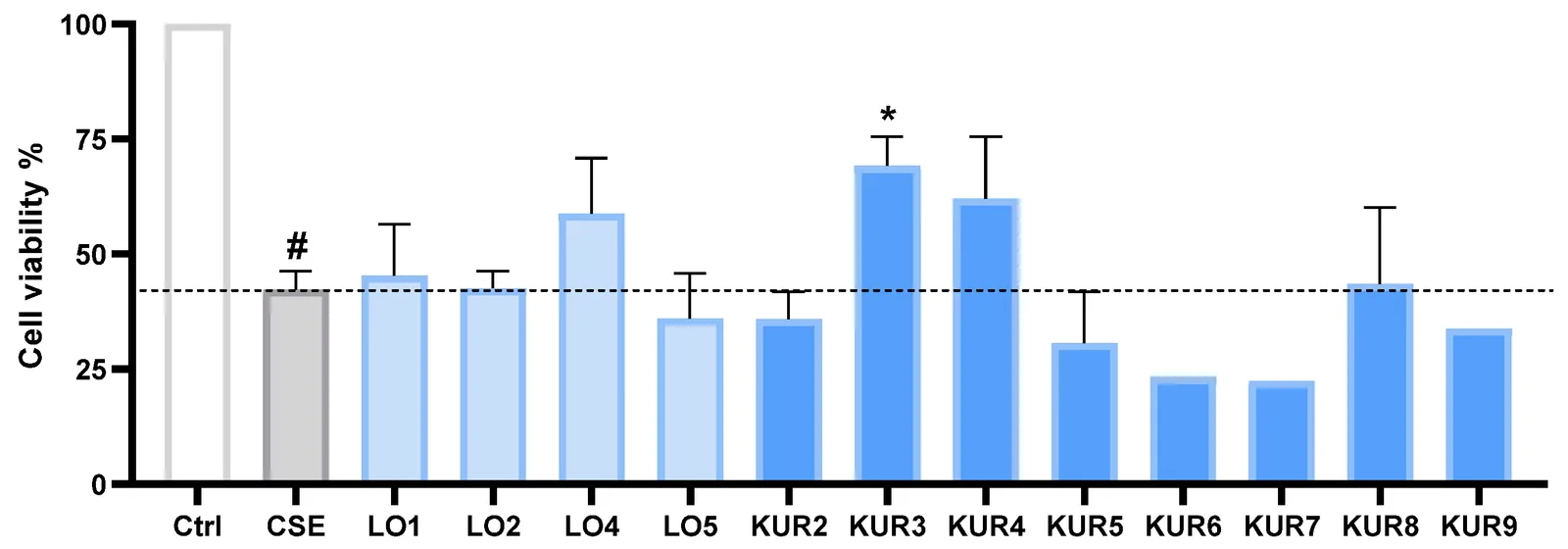
Cytoprotective effects of the isolated compounds against CSE-induced cytotoxicity in BEAS-2B cells. Cells were pretreated with the selected non-cytotoxic concentrations of the isolated compounds for 20 h and subsequently exposed to 20% CSE for 4 h. Cell viability was determined using the MTT assay. Ctrl: vehicle control (0.5% DMSO); CSE: cigarette smoke extract. Statistical significance was determined relative to the CSE group as * *p* < 0.05; CSE compared to Ctrl was indicated with # *p* < 0.05 (*n* = 3).

At the selected concentrations, the compounds were then evaluated for their ability to protect BEAS-2B cells against CSE-induced injury (Figure 3). Exposure to CSE reduced cell viability to 42.3% of the control value. Among the isolated compounds, ajugol (KUR3, 500 µM), aucubin (KUR4, 500 µM), and 1-*O*-caffeoyl-β-D-glucopyranose (LO4, 100 µM) produced the highest cell viability values, reaching 69.3%, 62.1%, and 58.8%, respectively. However, only ajugol produced a statistically significant cytoprotective effect compared with the CSE-treated group (*p* < 0.01). Luteolin 7-*O*-β-D-glucopyranoside (LO1) resulted in 45.4% cell viability and did not significantly protect against CSE-induced injury.

Based on the comparative cytoprotective screening, ajugol, aucubin, and 1-O-caffeoyl-β-D-glucopyranose, which produced the three highest cell viability values following CSE exposure, were prioritized for subsequent mechanistic evaluation. Although only ajugol reached statistical significance compared with the CSE-treated group, aucubin and 1-O-caffeoyl-β-D-glucopyranose showed the next most favorable activity profiles. The remaining isolated compounds, including verbascoside and harpagoside, were therefore not advanced to the subsequent mechanistic analyses.

### 2.4. Effects of Selected Compounds on CSE-Induced Inflammatory Responses

**Figure 4:**
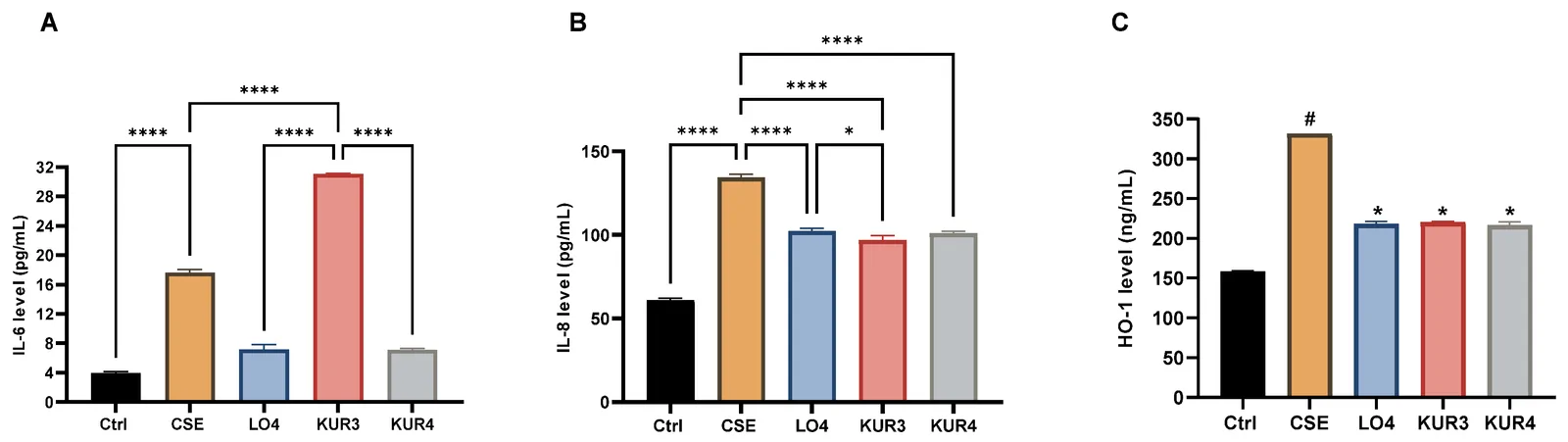
Effects of selected compounds on CSE-induced inflammatory and stress responses in BEAS-2B cells. (**A**) IL-6 levels, (**B**) IL-8 levels, and (**C**) HO-1 levels following pretreatment with 1-*O*-caffeoyl-β-D-glucopyranose (LO4), ajugol (KUR3), or aucubin (KUR4) for 20 h and subsequent exposure to 20% CSE for 4 h. Ctrl:, vehicle control (0.5% DMSO); CSE: cigarette smoke extract. Statistical significance is indicated as shown in the individual panels. In (**A**,**B**), group comparisons are indicated with * *p* < 0.05 and **** *p* < 0.01, whereas statistical comparisons of groups and Ctrl in (**C**) are indicated with # *p* < 0.05 and * *p* < 0.05 (*n* = 3).

The effects of the three compounds selected from the cytoprotective screening—1-*O*-caffeoyl-β-D-glucopyranose (LO4), ajugol (KUR3), and aucubin (KUR4)—were subsequently evaluated against CSE-induced inflammatory responses in BEAS-2B cells. Exposure to CSE for 4 h significantly increased the release of both IL-6 and IL-8 compared with the vehicle control (*p* < 0.001), confirming the induction of an acute inflammatory response under the experimental conditions (Figure 4A,B).

CSE exposure also significantly increased HO-1 levels compared with the control group (*p* < 0.001) (Figure 4C). Pretreatment with LO4, KUR3, and KUR4 significantly reduced the CSE-induced increase in HO-1 (*p* < 0.001), resulting in levels of 218.2 ± 4.72, 220.46 ± 1.21, and 216.72 ± 6.03 ng/mL, respectively.

MMP-9 was additionally examined as a marker associated with extracellular matrix remodeling. However, CSE exposure did not produce a significant increase in MMP-9 levels in either cell lysates or culture supernatants under the present experimental conditions. Consequently, compound-mediated inhibition of MMP-9 could not be meaningfully evaluated.

### 2.5. Effects of Selected Compounds on TLR4 Expression and Downstream Mediators

**Figure 5:**
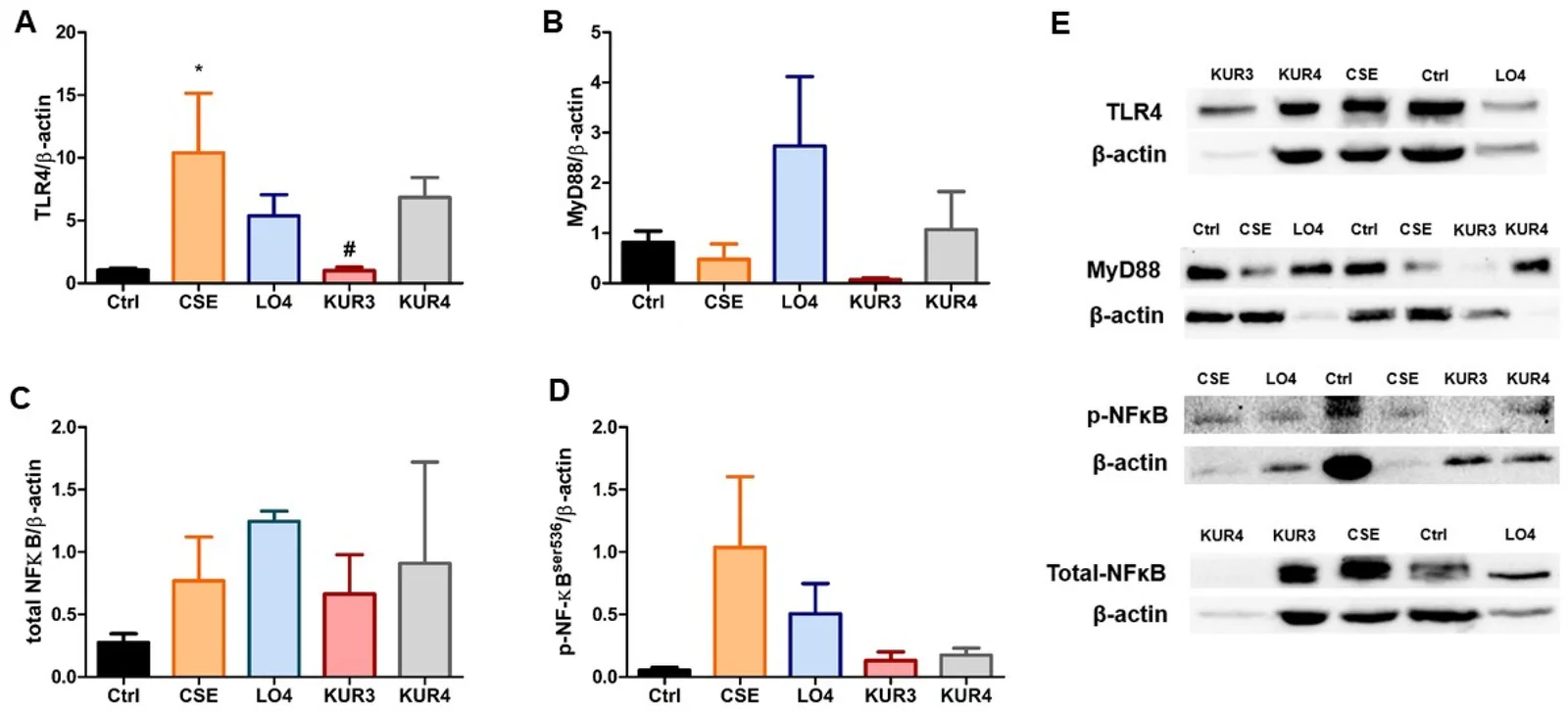
Effects of the selected compounds on CSE-induced TLR4/MyD88/NF-κB signaling in BEAS-2B cells. (**A**) TLR4 protein expression; (**B**) MyD88 protein expression; (**C**) total NF-κB protein expression; (**D**) p-NF-κB^Ser536^ protein expression; and (**E**) representative Western blot images of TLR4, MyD88, total NF-κB, and p-NF-κB^Ser536^ with β-actin as the loading control. BEAS-2B cells were pretreated with LO4 (1-*O*-caffeoyl-β-D-glucopyranose, 100 µM), KUR3 (ajugol, 500 µM), or KUR4 (aucubin, 500 µM) for 20 h and subsequently exposed to 20% CSE for 4 h. Data are expressed as mean ± SD. * *p* < 0.05 vs. control; # *p* < 0.05 vs. CSE. (*n* = 3).

To further investigate the molecular mechanisms underlying the anti-inflammatory effects of the selected compounds, the expression of TLR4, MyD88, total NF-κB, and phosphorylated NF-κB (p-NF-κB-Ser536) was evaluated in CSE-exposed BEAS-2B cells. Exposure to CSE significantly increased TLR4 protein expression compared with the untreated control (*p* < 0.05). Pretreatment with LO4 (1-*O*-caffeoyl-β-D-glucopyranose, 100 µM) or KUR4 (aucubin, 500 µM) did not significantly alter the CSE-induced increase in TLR4 expression. In contrast, KUR3 (ajugol, 500 µM) significantly reduced TLR4 expression compared with the CSE-treated group (*p* < 0.05), indicating that TLR4 modulation may contribute to the anti-inflammatory activity of ajugol (Figure 5A). In contrast to TLR4, MyD88 protein expression was not significantly altered by CSE exposure, and none of the three compounds produced a statistically significant change compared with the CSE-treated group (*p* > 0.05). Although an increase in MyD88 expression was observed in the 1-*O*-caffeoyl-β-D-glucopyranose group and a decrease was observed in the ajugol-treated group, these differences remained non-significant (Figure 5B). A similar pattern was observed for downstream NF-κB signaling. Total NF-κB protein expression did not differ significantly among the control, CSE, and compound-treated groups (*p* > 0.05). CSE exposure produced a tendency toward increased p-NF-κB-Ser536, whereas pretreatment with 1-*O*-caffeoyl-β-D-glucopyranose, ajugol, or aucubin was associated with lower p-NF-κB-Ser536 levels relative to the CSE group. However, none of these differences reached statistical significance (Figure 5C,D).

### 2.6. Effects of Selected Compounds on CSE-Induced Oxidative Stress

**Figure 6:**
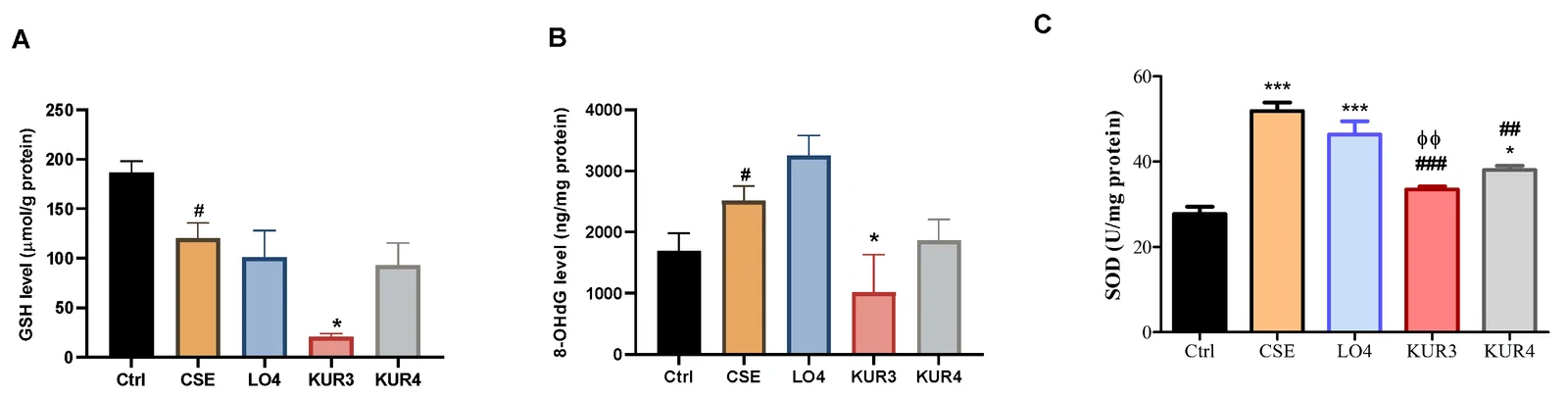
Effects of the selected compounds on CSE-induced oxidative stress in BEAS-2B cells. (**A**) Intracellular GSH levels. Statistical difference between Ctrl and CSE is indicated with # *p* < 0.01; CSE and group with * *p* < 0.05. (**B**) 8-OHdG levels as a marker of oxidative DNA damage. BEAS-2B cells were pretreated with LO4 (1-*O*-caffeoyl-β-D-glucopyranose, 100 µM), KUR3 (ajugol, 500 µM), or KUR4 (aucubin, 500 µM) for 20 h and subsequently exposed to 20% CSE for 4 h. Statistical difference between Ctrl and CSE is indicated with # *p* < 0.05; CSE vs. group with * *p* < 0.05. (**C**) Total SOD activity in the experimental groups. *** *p* < 0.001 and * *p* < 0.05 compared with the control group; ### *p* < 0.001 and ## *p* < 0.01 compared with the CSE group; ^ϕϕ^ *p* < 0.01 compared with LO4 indicates a statistically significant difference (*n* = 2).

Oxidative stress is a major component of CS-induced airway epithelial injury and contributes substantially to the pathogenesis of inflammatory lung diseases. To characterize the effects of the selected compounds on CSE-induced oxidative damage, intracellular glutathione (GSH) levels, superoxide dismutase (SOD) activity, and 8-hydroxy-2′-deoxyguanosine (8-OHdG) levels were evaluated in BEAS-2B cells. Our findings demonstrated that CSE exposure significantly depleted intracellular GSH in BEAS-2B cells (Figure 6A) (*p* < 0.01), whereas none of the compound pre-treatments restored the intracellular GSH pool. The GSH level in CSE-treated cells was 120.30 ± 21.7 µmol/g protein, whereas values of 101.13 ± 37.9, 20.69 ± 4.4, and 93.30 ± 31.2 µmol/g protein were measured following pretreatment with LO4 (1-*O*-caffeoyl-β-D-glucopyranose), KUR3 (ajugol), and KUR4 (aucubin), respectively.

The effects of the compounds on oxidative DNA damage showed a markedly different pattern. CSE exposure increased 8-OHdG levels from 1692.48 ± 407.5 ng/mg protein in control cells to 2514.42 ± 337.7 ng/mg protein (Figure 6B) (*p* < 0.05). Pretreatment with ajugol resulted in a pronounced reduction in 8-OHdG levels to 1014.8 ± 872.9 ng/mg protein, which was significantly lower than that observed in the CSE group (*p* < 0.05). Aucubin also produced a numerically lower 8-OHdG level (1861.62 ± 467.92 ng/mg protein), although a statistically significant difference from the CSE group was not reported. In contrast, 1-*O*-caffeoyl-β-D-glucopyranose did not reduce 8-OHdG levels, with a value of 3247.67 ± 489.72 ng/mg protein. Thus, among the three selected compounds, ajugol exhibited the clearest protective effect against CSE-associated oxidative DNA damage.

CSE exposure also significantly increased total SOD activity compared with the control (*p* < 0.001), indicating a marked alteration of the cellular oxidative response (Figure 6C). SOD activity remained significantly elevated in the 1-*O*-caffeoyl-β-D-glucopyranose -treated group compared with the control (*p* < 0.001), with no significant reduction relative to CSE alone. In contrast, ajugol significantly reduced SOD activity compared with the CSE group (*p* < 0.001) and produced significantly lower activity than 1-*O*-caffeoyl-β-D-glucopyranose (*p* < 0.01). Aucubin similarly reduced SOD activity relative to CSE (*p* < 0.01), although SOD activity remained significantly higher than the baseline control level (*p* < 0.05).

## 3. Discussion

The present study provides an integrated evaluation of the phytochemical composition and protective effects of *V. kurdicum* and *V. longipedicellatum* in a CSE-induced bronchial epithelial injury model. BEAS-2B cells provide a reproducible and widely used model for the initial investigation of CS-induced epithelial responses. However, their immortalized nature limits their ability to reproduce the differentiation status, barrier properties, variability, and physiological responses of primary cells of target organ. Therefore, the present findings represent preliminary screening-level evidence and should not be interpreted as demonstrating clinical efficacy. Within this frame, through bioactivity-guided fractionation, VK1A and VL1E were identified as the most promising fractions, and subsequent chromatographic purification yielded twelve structurally characterized compounds. Comparative biological evaluation of the isolated constituents revealed distinct activity profiles, with ajugol showing the most prominent overall protective effect, particularly in terms of cytoprotection, IL-8 suppression, attenuation of TLR4 upregulation, and reduction in oxidative DNA damage. In contrast, aucubin and 1-*O*-caffeoyl-β-D-glucopyranose displayed different patterns of activity, particularly in their effects on inflammatory cytokines. These findings indicate that the protective properties of the two *Verbascum* species are associated with chemically and functionally distinct constituents rather than with a single common mechanism. The findings are relevant in the context of CS-associated airway injury, as CSE has been shown to induce inflammatory and oxidative stress responses in human bronchial epithelial cells [35]. Moreover, elevated IL-6 levels have been reported in patients with COPD and have been associated with inflammatory burden, disease severity, and exacerbation status [36,37,38]. Taken together, the complementary activity profiles of VK1A and VL1E supported their selection for subsequent chromatographic isolation and identification of the bioactive constituents responsible for these effects.

The phytochemical profiles of the two active fractions were notably different. VL1E contained a relatively limited set of constituents, with luteolin 7-*O*-β-D-glucopyranoside as the major isolated component together with three minor compounds, consistent with the comparatively simple TLC profile observed for this fraction. In contrast, VK1A yielded chemically diverse constituents comprising iridoid glycosides, triterpenoid saponins, a phenylethanoid glycoside, and a nucleoside. This chemical diversity provided the basis for the subsequent comparative screening of the isolated compounds to identify the constituents contributing most strongly to the cytoprotective activity of the parent fractions.

The marked difference in cytoprotective efficacy among the isolated compounds indicates that the protective activity of the parent fractions cannot be attributed uniformly to all of their constituents. In particular, the superior response observed with ajugol suggests that the iridoid glycoside content of VK1A may contribute substantially to the cytoprotective activity detected during the initial fraction screening. Aucubin, another iridoid glycoside from the same active fraction, showed the second-highest viability value, although this effect did not reach statistical significance. In contrast, 1-*O*-caffeoyl-β-D-glucopyranose, isolated from VL1E, represented the most active constituent from *V. longipedicellatum* in this screening. These findings suggest that the cytoprotective effects of the two active fractions are likely driven by chemically distinct constituents rather than by a shared metabolite class. Because only ajugol showed statistically significant cytoprotection in the MTT assay, the effects observed for aucubin and 1-*O*-caffeoyl-β-D-glucopyranose should be regarded as comparatively favorable activity profiles rather than definitive cytoprotective effects at this stage.

The three compounds exhibited distinct effects on cytokine release. 1-*O*-caffeoyl-β-D-glucopyranose and aucubin significantly reduced CSE-induced IL-6 levels (*p* < 0.001), whereas ajugol did not significantly alter IL-6 release (Figure 4A). In contrast, all three compounds significantly suppressed CSE-induced IL-8 release, with ajugol producing the greatest reduction (Figure 4B). The effect of ajugol on IL-8 was also significantly greater than that observed with 1-*O*-caffeoyl-β-D-glucopyranose (*p* < 0.05). These findings indicate that the compounds differed in their cytokine-modulating profiles: aucubin and 1-*O*-caffeoyl-β-D-glucopyranose affected both IL-6 and IL-8 responses, whereas the anti-inflammatory profile of ajugol was more prominently associated with IL-8 suppression. Given the important contribution of IL-6 and IL-8 to CS-associated airway inflammation, these differential effects may reflect distinct actions of the compounds on inflammatory signaling rather than a uniform suppression of cytokine production.

The differential cytokine profiles observed for the selected compounds are supported, in part, by previous evidence for the anti-inflammatory activity of iridoid glycosides in respiratory models. Notably, aucubin has recently been shown to reduce both IL-6 and IL-8 levels in a CS-induced COPD model and to exert protective effects in CSE-exposed lung epithelial cells [39], consistent with the suppression of both cytokines observed in the present study. Ajugol, also referred to as leonuride, has likewise been reported to attenuate airway inflammation in an OVA-induced chronic asthma model, reducing inflammatory cell infiltration and type-2 cytokines and modulating transcriptional programs associated with cytokine/chemokine signaling and neutrophil chemotaxis [40]. Although IL-8 was not directly evaluated in that study, the reported regulation of chemotactic pathways is consistent with the pronounced effect of ajugol on IL-8 observed here. Together, these findings support the broader airway anti-inflammatory potential of ajugol and aucubin while also suggesting that their effects on individual cytokines may differ according to the inflammatory stimulus and cellular context. However, reduced HO-1 levels cannot independently be considered evidence of cytoprotection, as they may reflect either a reduction in upstream oxidative stress or suppression of the adaptive HO-1 response. This distinction is particularly relevant for 1-*O*-caffeoyl-β-D-glucopyranose, which reduced HO-1 despite persistently elevated SOD activity and 8-OHdG levels. In contrast, the parallel reductions in HO-1, SOD activity, and 8-OHdG observed with ajugol are more consistent with attenuation of the oxidative burden, although the underlying mechanism remains to be established. HO-1 is a stress-responsive protein with cytoprotective, antioxidant, and anti-inflammatory functions and is closely associated with Nrf2-mediated cellular responses to oxidative stress [41]. Accordingly, the increased HO-1 level following CSE exposure may represent an adaptive response to CSE-induced oxidative and inflammatory stress. The reduction observed after compound pretreatment should therefore not be interpreted simply as direct “inhibition” of HO-1; rather, together with the other protective responses observed in this study, it may indicate attenuation of the cellular stress stimulus responsible for HO-1 induction. Moreover, it should be noted that notable modulation of HO-1 may also contribute to the observed anti-inflammatory effects since it has an established role in the regulation of inflammatory signaling. This interpretation is consistent with previous studies describing the involvement of oxidative stress, Nrf2/HO-1 signaling, and inflammatory responses in CSE-exposed bronchial epithelial cells [1,35].

Interestingly, previous studies have reported activation or further enhancement of Nrf2/HO-1 signaling by aucubin in LPS-induced acute lung injury and CS-related COPD models [39,42]. In contrast, aucubin and the other selected compounds reduced the CSE-induced elevation of HO-1 in the present acute BEAS-2B model. This apparent difference suggests that the direction of HO-1 modulation may depend on the nature and duration of the inflammatory stimulus, cell type, and timing of assessment. Accordingly, the reduced HO-1 levels observed here should be interpreted in the context of the overall attenuation of CSE-induced cellular stress rather than as evidence of direct inhibition of the Nrf2/HO-1 pathway.

The absence of a detectable MMP-9 response may be related to the acute 4-h CSE exposure used in the present model, as MMP-9 has been associated with later stages of tissue injury and remodeling, whereas other matrix metalloproteinases may respond earlier during inflammatory and repair processes [43,44]. Thus, the present CSE model was sufficient to elicit marked cytokine and cellular stress responses but did not reproduce an MMP-9-associated remodeling response within the experimental exposure period.

The significant induction of TLR4 by CSE observed in the present study is consistent with previous evidence linking CS exposure to increased TLR4 signaling in airway epithelial cells. Geraghty et al. reported TLR4-dependent responses to CS in airway epithelial models, while Pace et al. observed increased TLR4 expression following CS exposure without a corresponding alteration in TLR2. Increased TLR4 expression has also been reported in smokers and shown to decrease following smoking cessation [45,46,47]. These observations support the biological relevance of the CSE-induced increase in TLR4 detected in our experimental model.

Among the three compounds, ajugol was distinguished by its ability to significantly attenuate CSE-induced TLR4 upregulation. This finding is particularly noteworthy when considered together with its pronounced inhibition of IL-8 described above, suggesting that modulation of early TLR4-associated inflammatory signaling may contribute to the anti-inflammatory profile of ajugol. However, because MyD88 expression and NF-κB phosphorylation were not significantly altered, the present findings do not establish inhibition of the complete canonical TLR4/MyD88/NF-κB pathway. Rather, they indicate a selective effect of ajugol at the level of TLR4 under the experimental conditions used. TLR4 signaling can proceed through both MyD88-dependent and MyD88-independent/TRIF-dependent pathways, with differences in the kinetics and downstream mediators involved [48,49,50]. Accordingly, the absence of significant changes in MyD88 despite altered TLR4 expression raises the possibility that additional TLR4-associated signaling mechanisms may contribute to the observed response. Nevertheless, TRIF-dependent signaling was not directly investigated in the present study, and therefore involvement of this pathway cannot be concluded from the current data.

Similarly, the absence of statistically significant changes in total NF-κB or p-NF-κB-Ser536 should be interpreted cautiously. NF-κB activation is highly dynamic, and the single 4-h CSE exposure endpoint used here may not fully capture transient changes in phosphorylation or nuclear translocation. Therefore, time-course experiments together with direct assessment of NF-κB nuclear translocation and additional components of MyD88-independent signaling would be required to define more precisely the downstream mechanism associated with ajugol-mediated TLR4 suppression.

Notably, the pronounced cytoprotective and anti-inflammatory effects previously observed for ajugol occurred despite the absence of GSH recovery, suggesting that restoration of the intracellular GSH pool is unlikely to account for its protective activity under these experimental conditions. Nevertheless, KUR3 and KUR4 pre-treatment reduced 8-OHdG levels relative to the CSE group, suggesting protection against oxidative DNA damage through a mechanism that was independent of GSH replenishment. In contrast, 1-*O*-caffeoyl-β-D-glucopyranose failed to attenuate either GSH depletion or 8-OHdG formation (Figure 6B).

The increased SOD activity following acute CSE exposure may reflect a cellular response to the increased oxidative burden. Accordingly, the reduction observed after ajugol and aucubin treatment could be consistent with attenuation of the CSE-associated oxidative stress response. However, reduced SOD activity alone cannot establish direct antioxidant or radical-scavenging activity, and this interpretation should therefore be considered together with the other CSE-induced oxidative stress parameters. Within this context, the marked reduction in 8-OHdG observed with ajugol provides complementary evidence that its modulation of SOD activity occurred alongside reduced oxidative DNA damage. By contrast, 1-*O*-caffeoyl-β-D-glucopyranose maintained elevated SOD activity and failed to reduce 8-OHdG, indicating that its previously observed anti-inflammatory activity was not accompanied by comparable protection against the oxidative endpoints evaluated here. This interpretation is also consistent with the overall assessment presented in the study report.

Importantly, the oxidative stress profile of aucubin in the present acute BEAS-2B model differs in some respects from previous findings. Liu et al. (2024) reported that aucubin increased SOD and GSH levels and reduced oxidative stress in CS-induced experimental inflammatory lung diseases, including CSE-exposed lung epithelial cells [39]. In the present model, aucubin reduced the CSE-associated increase in SOD activity but did not restore GSH. These apparently different responses may reflect differences in experimental model, exposure conditions, treatment concentration, or sampling time and indicate that changes in individual antioxidant enzymes should be interpreted in the context of the overall cellular redox response rather than as isolated indicators of antioxidant efficacy. It is important to note that our study employed an acute CSE-induced bronchial epithelial injury model for the initial screening of cytoprotective and anti-inflammatory effects. The 4-h exposure period was selected to generate a detectable cellular stress and inflammatory response while maintaining sufficient viability for evaluating treatment-related protection. Accordingly, this model was not designed to reproduce the chronic and multifactorial pathophysiology of inflammatory lung diseases. Furthermore, the use of a single immortalized bronchial epithelial cell line does not reflect the cellular heterogeneity, repeated smoke exposure, or immune–epithelial interactions involved in chronic respiratory disease. Hence, the findings should be interpreted as preliminary evidence obtained from an acute epithelial screening model rather than being directly extrapolated to COPD or other complex and multifactorial respiratory diseases. Further validation using repeated or prolonged CSE exposure, primary human airway epithelial cells, and advanced multicellular or three-dimensional airway models is warranted.

Taken together, the oxidative stress findings distinguish ajugol from the other selected compounds. Although none of the compounds restored depleted GSH, ajugol significantly reduced CSE-associated oxidative DNA damage and attenuated the CSE-induced increase in SOD activity. When considered together with its cytoprotective activity, suppression of IL-8, and reduction in TLR4 expression, these findings support a broader protective effect of ajugol against CSE-induced bronchial epithelial injury. Nevertheless, because intracellular ROS levels and direct radical-scavenging activity were not detected; thus, further studies incorporating direct intracellular ROS measurements and complementary oxidative damage endpoints are required to establish whether these effects involve direct antioxidant activity.

## 4. Materials and Methods

### 4.1. Phytochemical Studies

#### 4.1.1. Plant Material

The aerial parts of *V. kurdicum* and *V. longipedicellatum* were collected during flowering in July 2022 from Dikbayır Plateau (Dörtbölük Village) and Tinget Plateau (Sarıyayla Village), respectively, in Sason, Batman Province, Türkiye, by Assoc. Prof. Dr. Yeter Yeşil Cantürk (Istanbul University, Faculty of Pharmacy, Department of Pharmaceutical Botany). Air-dried and powdered aerial parts (370 and 300 g, respectively) were used for extraction. The species were identified by Assoc. Prof. Dr. Yeter Yeşil Cantürk, and voucher specimens were prepared and deposited in the Herbarium of the Faculty of Pharmacy, Istanbul University (ISTE), under the voucher numbers ISTE117826 for *V. kurdicum* and ISTE117827 for *V. longipedicellatum*.

#### 4.1.2. General Experimental Procedures

Thin-Layer Chromatography (TLC) was carried out on pre-coated silica gel *F*_254_ and reversed-phase *C*_18_ aluminum plates (Merck, Darmstadt, Germany) to monitor the fractions and control the purity of isolates. Developing solvent systems consisted of various ratios of CH_2_Cl_2_:MeOH:H_2_O (90:10:1 to 61:32:7, *v*/*v*/*v*) and *n*-hexane:EtOAc (9:1 to 1:2, *v*/*v*). Visualization of the TLC plates was performed under UV light (254 nm and 366 nm) and by spraying with 1% vanillin/H_2_SO_4_ reagent followed by heating at 105 °C for 3 min. Column chromatography was executed using polyamide (50–160 µm), silica gel 60 (0.063–0.200 mm, 70–230 mesh, Merck), and Sephadex LH-20 (GE Healthcare, Munich, Germany). Vacuum Liquid Chromatography (VLC) and Medium-Pressure Liquid Chromatography (MPLC) were conducted using LiChroprep^®^ RP-18 (40–63 µm, Merck) stationary phase. The MPLC system consisted of a Buchi Sepacore pump module, fraction collector (Buchi C-660, Buchi, Flawil, Switzerland), and prepacked columns (4 g and 12 g). Structural elucidation was performed using 1D and 2D Nuclear Magnetic Resonance (NMR) spectroscopy; ^1^H-NMR (400 MHz) and ^13^C-NMR (100 MHz) spectra were recorded on a Bruker AVANCE III spectrometer (Bruker, Karlsruhe，Germany) in deuterated solvents.

#### 4.1.3. Extraction and Fractionation

The powdered aerial parts of *V. kurdicum* (370 g) and *V. longipedicellatum* (300 g) were separately extracted with 50% methanol (MeOH) at 40 °C for 8 h. The extraction procedure was repeated four times using fresh solvent, and the resulting filtrates were combined and concentrated to dryness under reduced pressure at 40 °C using a rotary evaporator. The extraction yielded 70.81 g (19.14%) and 55.91 g (18.64%) of crude MeOH extracts from *V. kurdicum* and *V. longipedicellatum*, respectively. Approximately 1 g of each crude extract was reserved for biological activity assays. The remaining extracts were dissolved in MeOH:H_2_O (1:9) and sequentially partitioned with *n*-hexane and *n*-butanol (*n*-BuOH), with each partitioning step repeated three times. After solvent removal, *V. kurdicum* yielded *n*-hexane (0.90 g), *n*-BuOH (36.83 g), and aqueous (29.95 g) fractions, whereas *V. longipedicellatum* yielded *n*-hexane (0.91 g), *n*-BuOH (28.41 g), and aqueous (24.80 g) fractions.

#### 4.1.4. Isolation of Compounds from *V. longipedicellatum*

The *n*-BuOH extract of *V. longipedicellatum* was subjected to polyamide column chromatography (3.2 × 46 cm, 100 g polyamide) using an H_2_O-MeOH gradient (100:0→0:100, *v*/*v*), yielding 25 fractions. Fractions with similar TLC profiles were combined into six pooled fractions (VL1A-VL1F), of which VL1E (1.13 g) was selected for further purification based on its cytoprotective activity. VL1E was subjected to vacuum liquid chromatography (VLC) over LiChroprep^®^ RP-18 using a stepwise MeOH-H_2_O gradient (0:100→100:0, *v*/*v*). VLC fractions 36–39, 87–94, 95–97, 98–100, 101–105, and 106 were individually purified by Sephadex LH-20 column chromatography using MeOH as the eluent, yielding LO-1 (luteolin 7-*O*-β-D-glucopyranoside, 24.2 mg). VLC fractions 62–86 were further separated by silica gel 60 column chromatography, and the resulting fractions were combined according to their TLC profiles. Selected pooled fractions (178–190 and 201–206) were subsequently purified by Sephadex LH-20 column chromatography followed by RP-18 MPLC, affording LO-2 (ethyl 2,4-dihydroxybutanoate, 1.0 mg) and LO-4 (1-*O*-caffeoyl-β-D-glucopyranose, 4.7 mg). LO-5 (*p*-anisaldehyde, 7.4 mg) was also isolated from the active VL1E fraction.

#### 4.1.5. Isolation of Compounds from *V. kurdicum*

The *n*-BuOH extract of *V. kurdicum* was subjected to polyamide column chromatography (3.2 × 46 cm, 100 g polyamide) using a H_2_O-MeOH gradient (100:0→0:100, *v*/*v*), yielding 35 fractions. Fractions with similar TLC profiles were combined into five pooled fractions: VK1A (7.52 g), VK1B (3.78 g), VK1C (2.68 g), VK1D (2.21 g), and VK1E (0.94 g). Based on bioactivity-guided screening, VK1A, which exhibited the highest activity among the fractions, was selected for further purification.

VK1A (7.52 g) was subjected to vacuum liquid chromatography (VLC) over LiChroprep^®^ RP-18 using a stepwise MeOH:H_2_O gradient (0:100→100:0, *v*/*v*). VLC fractions 34–51 were purified by Sephadex LH-20 column chromatography using 100% MeOH to yield KUR-2 (adenosine, 2.4 mg). Fractions 34–64 obtained from the same Sephadex LH-20 column were subsequently subjected to silica gel 60 open-column chromatography to afford KUR-3 (ajugol, 46 mg). VLC fractions 19–28 were directly purified by RP-18 MPLC to yield KUR-4 (aucubin, 3.9 mg). In a separate purification pathway, VLC fractions 96–99 were subjected to silica gel 60 column chromatography. The resulting subfractions 331–354 were further purified by RP-18 MPLC to afford KUR-5 (ilwensisaponin C, 7.8 mg), KUR-6 (songarosaponin E, 4.1 mg), and KUR-7 (ilwensisaponin A, 4.0 mg), while subfractions 236–244 obtained from the same silica gel column were purified by RP-18 MPLC to yield KUR-9 (harpagoside, 3.3 mg). VLC fractions 86–87 were subjected to silica gel 60 column chromatography, and the resulting subfractions 63–65 were further purified by RP-18 MPLC to yield KUR-8 (verbascoside, 2.7 mg).

#### 4.1.6. Spectroscopic Analysis

The structures of the isolated compounds were identified using one- and two-dimensional NMR spectroscopy. ^1^H and ^13^C NMR spectra were recorded on a Bruker AVANCE III spectrometer operating at 400 and 100 MHz, respectively. Additional NMR experiments, including APT, COSY, HSQC, and HMBC, were performed as appropriate. Structural assignments were made by interpretation of the spectroscopic data and comparison with previously reported literature data.

### 4.2. Biological Evaluation

#### 4.2.1. Preparation of Cigarette Smoke Extract (CSE)

Cigarette smoke extract (CSE) was prepared according to a previously optimized method [1,2]. Briefly, two research cigarettes (University of Kentucky, USA) were burned, and the smoke was bubbled through 6 mL of serum-free cell culture medium. The resulting extract was filtered through a 0.22 µm membrane, and fetal bovine serum (FBS; Gibco, Grand Island, NY, USA) was added to a final concentration of 5% (*v*/*v*). The resulting solution was designated as 100% CSE, aliquoted, and stored at −80 °C until use. Prior to experiments, the CSE stock was diluted with DMEM High Glucose medium (Gibco, USA) to the required working concentration.

#### 4.2.2. Cell Culture

The human bronchial epithelial cell line BEAS-2B (American Type Culture Collection, ATCC, USA) was cultured in Dulbecco’s Modified Eagle Medium High Glucose (DMEM High Glucose; Gibco, USA) supplemented with 5% (*v*/*v*) FBS and 100 IU/mL penicillin and streptomycin solution. Cells were maintained at 37 °C in a humidified atmosphere containing 5% CO_2_ and passaged using 0.25% trypsin upon reaching approximately 80% confluence, as previously described [1].

#### 4.2.3. Cytotoxicity and Cytoprotective Assays (MTT)

Cell viability was evaluated using the 3-(4,5-dimethylthiazol-2-yl)-2,5-diphenyltetrazolium bromide (MTT) assay, based on a previously modified protocol [1]. BEAS-2B cells were seeded into 48-well plates at a density of 30,000 cells/well and allowed to adhere overnight. For cytotoxicity evaluation, the fractions were tested at five concentrations within 0.01–1 mg/mL, whereas the isolated compounds were evaluated over compound-specific concentration ranges selected according to solubility and preliminary testing, spanning 0.01–500 µM. The highest concentration maintaining cell viability at or above 70% relative to the negative control was considered non-cytotoxic and selected for subsequent cytoprotective experiments.

For cytoprotective evaluation, cells were pre-incubated with the selected non-cytotoxic concentrations of the fractions or compounds for 20 h, followed by CSE exposure for an additional 4 h. After a total incubation period of 24 h, the culture medium was removed, and 100 µL of MTT solution (0.5 mg/mL) was added to each well. Cells were incubated for 2 h at 37 °C under 5% CO_2_, after which the resulting formazan crystals were dissolved in isopropanol. Absorbance was measured at 570 nm using a Thermo Varioskan microplate reader (Thermo Fisher Scientific, Singapore). Relative cell viability was calculated as a percentage of the negative/solvent vehicle control, which received 0.5% (*v*/*v*) DMSO, at a negligible concentration for biological response. All measurements were performed in triplicate.

#### 4.2.4. Enzyme-Linked Immunosorbent Assays (ELISA)

Cell culture supernatants obtained after CSE and compound treatments were used for the quantitative determination of IL-6, IL-8, HO-1, and MMP-9 using commercially available human ELISA kits (Elabscience Biotechnology Co., Ltd. Houston, TX, USA). IL-6 and IL-8 levels were measured using the Human IL-6 ELISA Kit (E-EL-H6156) and Human IL-8 ELISA Kit (E-EL-H6008), respectively. Supernatants were diluted 1:5 (*v*/*v*), and 50 µL of each diluted sample was added to the corresponding wells. After incubation at 37 °C for 30 min, plates were washed five times, followed by incubation with 100 µL HRP conjugate at 37 °C for 30 min. After a second washing step, 90 µL substrate solution was added and incubated at 37 °C for 15 min in the dark. The reaction was terminated with 50 µL stop solution, and absorbance was measured at 450 nm using a Thermo Varioskan microplate reader. IL-6 and IL-8 concentrations were calculated from calibration curves prepared over the ranges of 1.56–100 pg/mL and 7.81–500 pg/mL, respectively, and corrected for the dilution factor. Samples were analyzed in duplicate.

HO-1 and MMP-9 levels were determined using the Human HO-1 ELISA Kit (E-EL-H2172) and Human MMP-9 ELISA Kit (E-EL-H6075), respectively. For both assays, 100 µL of samples, blanks, and standards was added to the wells and incubated at 37 °C for 90 min. After removal of the well contents, 100 µL biotin conjugate was added and incubated for 1 h at 37 °C. The wells were washed three times, followed by incubation with 100 µL HRP conjugate for 30 min at 37 °C. After five additional washing cycles, 90 µL substrate solution was added and incubated for 15 min in the dark. The reaction was stopped with 50 µL stop solution, and absorbance was measured at 450 nm. Results were expressed as ng/mL, and all samples were analyzed in duplicate.

#### 4.2.5. Western Blot Analysis

BEAS-2B cell pellets were incubated in ice-cold TNTE lysis buffer for 15 min and homogenized using sonication and repeated pipetting. The homogenates were centrifuged at 13,000 rpm for 15 min at 4 °C, and the resulting supernatants were collected. Total protein concentrations were determined using a bicinchoninic acid (BCA) protein assay kit (Thermo Scientific, Waltham, MA, USA). Protein samples were mixed with 5× Laemmli buffer containing 0.25 M Tris-HCl (pH 6.8), 10% SDS, 50% glycerol, 0.01% bromophenol blue, and 0.5 mM dithiothreitol (DTT), and denatured at 95 °C for 5 min. Equal amounts of protein (30 µg) were separated on 10% polyacrylamide gels using SDS-containing Tris/glycine running buffer at 100 V and transferred onto polyvinylidene fluoride (PVDF) membranes by wet transfer at 80 V for 2 h, as previously described [51].

Membranes were stained with 0.1% Ponceau S in 5% acetic acid to verify protein transfer, washed with TBST containing 0.1% Tween-20, and blocked with 5% non-fat dry milk in TBST for 90 min at room temperature. Membranes were then incubated overnight with primary antibodies against TLR4 (Santa Cruz Biotechnology, Dallas, TX, USA, sc-293072, 1:1000), MyD88 (Cell Signaling Technology, Danvers, MA, USA, #4283, 1:1000), total NF-κB (Cell Signaling Technology, Danvers, MA, USA, #8242, 1:1000), and phospho-NF-κB Ser536 (Cell Signaling Technology, Danvers, MA, USA, #3033, 1:1000) in TBST containing 3% BSA. After washing, membranes were incubated with HRP-linked anti-rabbit IgG secondary antibody (Cell Signaling Technology, Danvers, MA, USA, #7074, 1:5000) for 1 h at room temperature. β-Actin (Cell Signaling Technology, Danvers, MA, USA, #3700, 1:3000) was used as the loading control. Protein bands were visualized using enhanced chemiluminescence with Clarity Max Western ECL Substrate (Bio-Rad, Hercules, CA, USA), imaged using a ChemiDoc MP system (Bio-Rad), and quantified using ImageJ software (U. S. National Institutes of Health, Bethesda, MD, USA).

#### 4.2.6. Measurement of Oxidative Stress Biomarkers

Intracellular glutathione (GSH) levels were determined using a previously optimized colorimetric method [1]. The absorbance of the yellow chromophore formed in proportion to GSH concentration was measured at 412 nm. Phosphate buffer was used as the blank, and serial dilutions of a GSH stock solution (Sigma-Aldrich, Darmstadt, Germany; 0.125–2 mM) were used for calibration. GSH levels were normalized to total protein content and expressed as µmol/g protein. Measurements were performed in triplicate.

Oxidative DNA damage was assessed by measuring 8-hydroxy-2′-deoxyguanosine (8-OHdG) using a commercial ELISA kit (Elabscience, Wuhan, China, E-EL-0028). BEAS-2B cells were seeded in 24-well plates at a density of 4 × 10^4^ cells/well and treated with the selected compounds and CSE under the conditions established in the cytoprotective assays. Following incubation, cell supernatants were collected and analyzed according to the manufacturer’s protocol. Briefly, 50 µL of standards (1.56–100 ng/mL) or samples was added to the wells, followed by 25 µL of 8-OHdG conjugate and, except for the non-specific binding wells, 25 µL of 8-OHdG antibody. Plates were incubated for 2 h at room temperature on a shaker at 300 rpm, washed four times, and incubated with 100 µL TMB substrate for 30 min. The reaction was terminated with 50 µL stop solution, and absorbance was measured at 450 nm. 8-OHdG concentrations were calculated from the standard curve.

Total superoxide dismutase (SOD) activity was determined using a WST-1-based commercial assay kit (Elabscience, Wuhan, China E-BC-K020-M, Total SOD Activity Assay Kit) according to the manufacturer’s instructions. Approximately 1 × 10^6^ cells from each experimental group were collected, washed with ice-cold 0.01 M phosphate-buffered saline (PBS, pH 7.4), resuspended in 500 µL PBS, and lysed by ultrasonic homogenization on ice. Cell homogenates were centrifuged at 10,000× *g* for 10 min at 4 °C, and the resulting supernatants were used for SOD activity determination. Total SOD activity was calculated from the percentage inhibition according to the manufacturer’s formula, normalized to total protein concentration determined by the BCA method, and expressed as U/mg protein.

#### 4.2.7. Statistical Analysis

Statistical analyses were performed using GraphPad Prism 8.0 (GraphPad Software, San Diego, CA, USA). Data from cytotoxicity assays, SOD and GSH measurements, ELISA analyses, and Western blot densitometry are presented as the mean ± standard deviation (SD) of independent experiments. Technical replicates within each independent experiment were averaged before statistical analysis and were not treated as independent observations. The number of independent experiments (*n*) is specified in the corresponding figure legends. Data distribution was assessed using the Shapiro–Wilk test. Comparisons between two groups were performed using an unpaired two-tailed Student’s *t*-test for normally distributed data or the Mann–Whitney *U* test for non-normally distributed data. For comparisons involving more than two groups, one-way ANOVA followed by Bonferroni’s multiple-comparisons test was used when the assumptions of parametric analysis were satisfied. Non-normally distributed multiple-group data were analyzed using the Kruskal–Wallis test followed by Dunn’s multiple-comparisons test. The same statistical approach was applied to normalized Western blot densitometry data. *p*-value < 0.05 was considered statistically significant.

## 5. Conclusions

This study provides the first integrated phytochemical and preliminary biological investigation of *V. kurdicum* and *V. longipedicellatum* and links their chemical constituents to cytoprotective activity in a CSE-induced acute bronchial epithelial injury model. Bioactivity-guided fractionation identified VK1A and VL1E as the principal active fractions and led to the isolation of twelve structurally characterized compounds representing several natural-product classes. Comparative biological screening further identified ajugol, aucubin, and 1-*O*-caffeoyl-β-D-glucopyranose as the compounds with the most prominent activity profiles.

The three selected compounds displayed clearly differentiated biological effects. Aucubin and 1-*O*-caffeoyl-β-D-glucopyranose preferentially suppressed CSE-induced IL-6 release, whereas ajugol showed a particularly pronounced effect on IL-8 and modulated TLR4 expression in the BEAS-2B model exposed to CSE, rather than presenting evidence for complete inhibition of the entire TLR4/MyD88/NF-κB pathway. Ajugol also provided the clearest protection against oxidative DNA damage, significantly reducing 8-OHdG levels despite the absence of GSH restoration. Together with its significant cytoprotective activity and modulation of the CSE-induced SOD response, these findings identify ajugol as the compound exhibiting the broadest protective profile among the isolated metabolites.

Importantly, the absence of significant changes in MyD88 and NF-κB phosphorylation and the divergent responses observed among GSH, SOD, and 8-OHdG indicate that the protective effects cannot be attributed to a single canonical inflammatory or oxidative pathway. Rather, the results suggest compound-specific and potentially multi-target mechanisms that require further clarification. Time-dependent signaling studies, direct measurements of intracellular ROS, assessment of NF-κB nuclear translocation and MyD88-independent TLR4 signaling, and validation using chronic or repeated-exposure in vitro models and advanced three-dimensional lung systems will be required to further elucidate the underlying mechanisms. Appropriately justified in vivo studies may subsequently be considered to evaluate the pharmacological relevance of these findings.

Overall, the findings demonstrate that these previously unexplored *Verbascum* species represent valuable sources of bioactive secondary metabolites and provide a rationale for further investigation of ajugol and aucubin in CS-associated inflammatory and oxidative lung injury.

## Data Availability

The data supporting the findings of this study are provided within the article. Additional information is available from the corresponding author upon reasonable request.

## Supplementary Materials

The following supporting information can be downloaded at: https://www.mdpi.com/article/10.3390/molecules31213818/s1, Figure S1: Cytotoxicity profiles of *Verbascum kurdicum* extracts and fractions in BEAS-2B cells; Figure S2: Cytotoxicity profiles of *Verbascum longipedicellatum* extracts and fractions in BEAS-2B cells; Figure S3: NMR spectra of LO-1 (luteolin 7-*O*-β-D-glucopyranoside); Figure S4: NMR spectra of LO-2 (ethyl 2,4-dihydroxybutanoate); Figure S5: NMR spectra of LO-4 (1-*O*-caffeoyl-β-D-glucopyranose); Figure S6: ^1^H NMR spectrum of LO-5 (*p*-anisaldehyde); Figure S7: NMR spectra of KUR-2 (adenosine); Figure S8: NMR spectra of KUR-3 (ajugol); Figure S9: NMR spectra of KUR-4 (aucubin); Figure S10: NMR spectra of KUR-5 (ilwensisaponin C); Figure S11: NMR spectra of KUR-6 (songarosaponin E); Figure S12: NMR spectra of KUR-7 (ilwensisaponin A); Figure S13: NMR spectra of KUR-8 (verbascoside); Figure S14: NMR spectra of KUR-9 (harpagoside); Figure S15: HPLC-DAD chromatograms of selected isolated compounds used for chromatographic purity assessment; Figure S16: Cytotoxicity profiles of the isolated compounds in BEAS-2B cells; Table S1: Non-cytotoxic concentrations of *V. kurdicum* and *V. longipedicellatum* extracts and fractions selected for cytoprotective screening in BEAS-2B cells; Table S2: HPLC-DAD chromatographic purity of selected isolated compounds determined by peak-area normalization at 254 nm; Table S3: Concentrations of the isolated compounds selected for cytoprotective screening in BEAS-2B cells.

## Abbreviations

The following abbreviations are used in this manuscript:

8-OHdG	8-hydroxy-2′-deoxyguanosine
BEAS-2B	Bronchial Epithelium transformed with Adenovirus 12-SV40 2B
COPD	Chronic obstructive pulmonary disease
CS	Cigarette smoke
CSE	Cigarette smoke extract
GSH	Glutathione
HO-1	Heme oxygenase-1
IL-6	Interleukin-6
IL-8	Interleukin-8
MyD88	Myeloid differentiation primary response 88
NF-κB	Nuclear factor-kappa B
NMR	Nuclear magnetic resonance
SOD	Superoxide dismutase
TLR4	Toll-like receptor 4
