# Supplementary material for: Bioactivity-Guided Isolation of Cytoprotective Metabolites from Two Verbascum Species Reveals Differential Modulation of Cigarette Smoke-Induced Inflammation and Oxidative Stress
Source: Molecules. 2026 Oct 22;31(21):3818. doi: 10.3390/molecules31213818 (PMC13765239; doi:10.3390/molecules31213818)
Supplement: Supplementary file 1 [file molecules-31-03818-s001.zip › molecules-4568566-supplementary.pdf]

**SUPPLEMENTARY MATERIAL**

# Bioactivity-Guided Isolation of Cytoprotective Metabolites from Two *Verbascum* Species Reveals Differential Modulation of Cigarette Smoke-Induced Inflammation and Oxidative Stress

Gülin Renda,[a,b] Nurdan Yazıcı,[a] Rengin Reis,[c] Elif Nur Barut,[d] Burak Barut,[e] İçim Gökkaya,[f,g] Elif Öz,[c] Manar M. Amin,[h] Yeter Yeşil Cantürk,[i]

[a]*Department of Pharmacognosy, Faculty of Pharmacy, Karadeniz Technical University, Trabzon, Türkiye*

[b]*Trabzon Teknokent, Farmakodental Health Products, Trabzon, Türkiye*

[c]*Department of Toxicology, Faculty of Pharmacy, Acibadem Mehmet Ali Aydinlar University, Istanbul, Türkiye*

[d]*Department of Pharmacology, Faculty of Pharmacy, Karadeniz Technical University, Trabzon, Türkiye*

[e]*Department of Biochemistry, Faculty of Pharmacy, Karadeniz Technical University, Trabzon, Türkiye*

[f]*Department of Pharmaceutical Botany, Faculty of Pharmacy, Karadeniz Technical University, Trabzon, Türkiye*

[g]*Institute of Health Sciences, Ankara University, Ankara, Türkiye*

[h]*Department of Pharmacology, Graduate School of Health Sciences, Karadeniz Technical University, Trabzon, Türkiye*

[i]*Department of Pharmaceutical Botany, Faculty of Pharmacy, Istanbul University, Istanbul, Türkiye*

**Figure S1. Cytotoxicity profiles of *Verbascum kurdicum* extracts and fractions in BEAS-2B cells.**

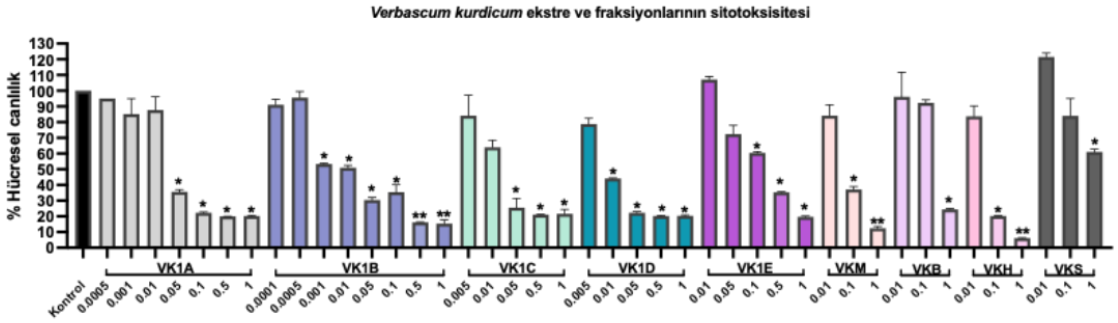


BEAS-2B cells were exposed to the extracts and fractions for 24 h, and cell viability was assessed using the MTT assay. Control, 0.5% (v/v) DMSO; VKM, *V. kurdicum* methanol extract; VKB, V. kurdicum n-butanol extract; VKH, *V. kurdicum n*-hexane extract; VKS, *V. kurdicum* aqueous extract; VK1A-VK1E, fractions obtained from the *n*-butanol extract. Statistical significance versus the control group: **p* < 0.05 and ***p* < 0.01.

**Figure S2. Cytotoxicity profiles of *Verbascum longipedicellatum* extracts and fractions in BEAS-2B cells.**

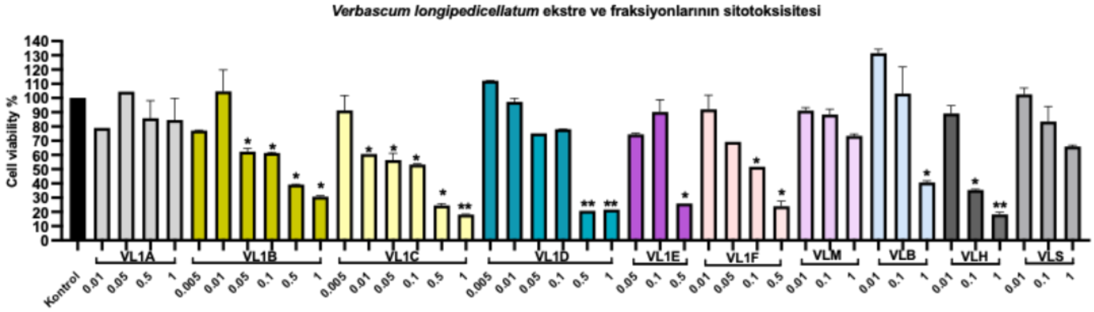


BEAS-2B cells were exposed to the extracts and fractions for 24 h, and cell viability was assessed using the MTT assay. Control, 0.5% (v/v) DMSO; VLM, *V. longipedicellatum* methanol extract; VLB, *V. longipedicellatum n*-butanol extract; VLH, *V. longipedicellatum n*-hexane extract; VLS, *V. longipedicellatum* aqueous extract; VL1A-VL1F, fractions obtained from the *n*-butanol extract. Statistical significance versus the control group: **p* < 0.05 and ***p* < 0.01.

**Table S1. Non-cytotoxic concentrations of *V. kurdicum* and *V. longipedicellatum* extracts and fractions selected for cytoprotective screening in BEAS-2B cells.**

| Species | Sample | Extract/fraction | Selected concentration (mg/mL) |
|---|---|---|---|
| *V. kurdicum* | VKM | Methanol extract | 0.01 |
| | VKB | *n*-Butanol extract | 0.01 |
| | VK1A | Polyamide fraction | 0.01 |
| | VK1B | Polyamide fraction | 0.0005 |
| | VK1C | Polyamide fraction | 0.005 |
| | VK1D | Polyamide fraction | 0.005 |
| | VK1E | Polyamide fraction | 0.05 |
| *V. longipedicellatum* | VLM | Methanol extract | 0.1 |
| | VLB | *n*-Butanol extract | 0.1 |
| | VL1A | Polyamide fraction | 0.5 |
| | VL1B | Polyamide fraction | 0.01 |
| | VL1C | Polyamide fraction | 0.005 |
| | VL1D | Polyamide fraction | 0.01 |
| | VL1E | Polyamide fraction | 0.1 |
| | VL1F | Polyamide fraction | 0.01 |

**LO-1 (luteolin 7-*O*-β-D-glucopyranoside).** The $^{1}$H NMR spectrum showed characteristic signals of a luteolin-type flavonoid, including aromatic proton resonances at $\delta_H$ 7.41, 7.38, 6.87, 6.77, 6.58, and 6.47 ppm. The anomeric proton signal at $\delta_H$ 5.03 (d, $J$ = 7.23 Hz) supported the presence of a β-configured glucopyranosyl unit. In the HMBC spectrum, the correlation between the anomeric proton at $\delta_H$ 5.03 and the carbon resonance at $\delta_C$ 160.8 supported glycosylation at C-7 of the flavonoid aglycone. Comparison of the spectroscopic data with previously reported values confirmed the structure as luteolin 7-*O*-β-D-glucopyranoside (Donia, 2016) (Figure S3).

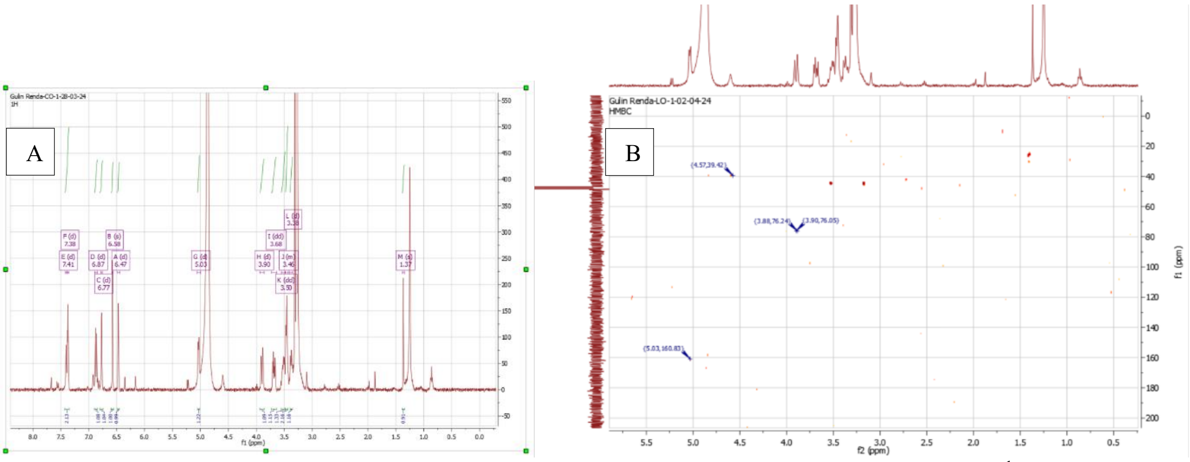


**Figure S3. NMR spectra of LO-1 (luteolin 7-*O*-β-D-glucopyranoside).** (A) $^{1}$H NMR spectrum; (B) HMBC spectrum.

**LO-2 (ethyl 2,4-dihydroxybutanoate).** The $^{1}$H NMR spectrum showed characteristic signals of an aliphatic ethyl ester, including resonances at $\delta_H$ 4.00 (q) and 1.32 ppm attributable to the ethoxy group. A multiplet at $\delta_H$ 4.09 was assigned to the oxygenated methine proton, while signals in the $\delta_H$ 3.45–3.92 ppm region were consistent with the terminal hydroxymethylene group. The $^{13}$C-APT spectrum further supported the presence of oxygenated aliphatic carbons, with resonances at $\delta_C$ 72.46 and 63.1 ppm, together with the ethoxy methylene carbon at $\delta_C$ 60.5 ppm and an aliphatic methylene carbon at $\delta_C$ 19.7 ppm. In the COSY spectrum, the cross-peak between $\delta_H$ 1.32 and 4.00 confirmed the ethoxy spin system, while sequential proton correlations supported the butanoate chain. The spectroscopic data were consistent with those previously reported for ethyl 2,4-dihydroxybutanoate (Chang et al., 2009) (Figure S4).

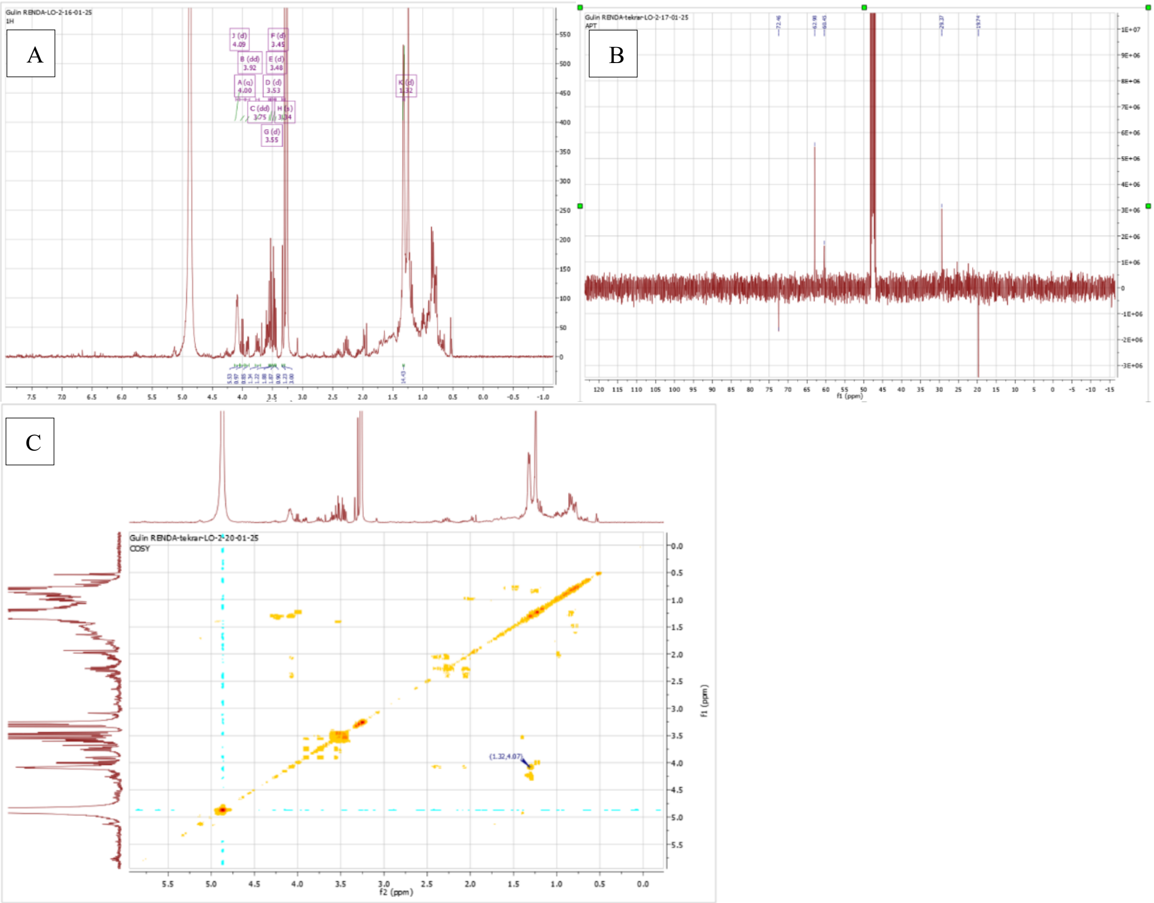


**Figure S4. NMR spectra of LO-2 (ethyl 2,4-dihydroxybutanoate).** (A) $^{1}$H NMR spectrum; (B) $^{13}$C-APT spectrum; (C) COSY spectrum.

**LO-4 (1-*O*-caffeoyl-β-D-glucopyranose).** The $^{1}$H NMR spectrum showed characteristic signals of a caffeoyl moiety, including trans-olefinic proton resonances at $\delta_H$ 7.55 and 6.23 ppm and an ABX-type aromatic proton system at $\delta_H$ 7.01, 6.82, and 6.73 ppm. The anomeric proton signal at $\delta_H$ 5.14, together with resonances in the $\delta_H$ 3.45-4.49 ppm region, supported the presence of a glucopyranosyl unit. The $^{13}$C-APT spectrum showed an ester carbonyl resonance at $\delta_C$ 166.11 and an anomeric carbon at $\delta_C$ 88.4, along with the expected oxygenated sugar carbons. Importantly, the HMBC correlation between the anomeric proton at $\delta_H$ 5.14 and the ester carbonyl carbon at $\delta_C$ 166.1 established the caffeoyl group at the C-1 position of glucose.

The spectroscopic data were consistent with those previously reported for 1-*O*-caffeoyl-β-D-glucopyranose (Thi et al., 2017; Li et al., 2021) (Figure S5).

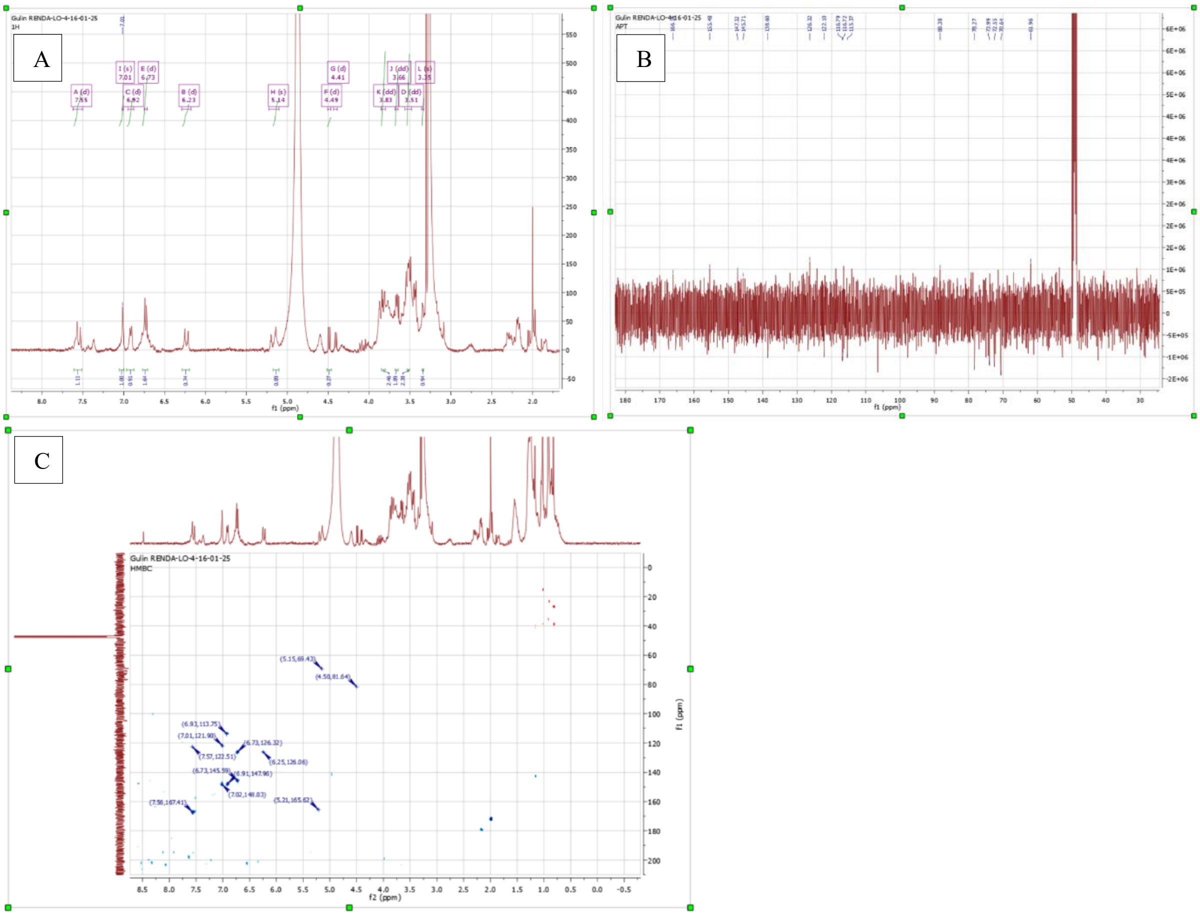


**Figure S5. NMR spectra of LO-4 (1-*O*-caffeoyl-β-D-glucopyranose).** (A) $^{1}$H NMR spectrum; (B) $^{13}$C-APT spectrum; (C) HMBC spectrum.

**LO-5 (*p*-anisaldehyde).** The $^{1}$H NMR spectrum showed a characteristic aldehyde proton signal at δ_H 9.82 (s), together with two aromatic doublets at $\delta_H$ 7.78 ($J$ = 8.8 Hz) and 6.94 ($J$ = 8.7 Hz), consistent with an AA'BB' spin system of a *para*-disubstituted aromatic ring. A singlet at $\delta_H$ 3.83 was attributable to the methoxy group. The spectroscopic data were consistent with those previously reported for *p*-anisaldehyde (4-methoxybenzaldehyde) (Islam et al., 2012) (Figure S6).

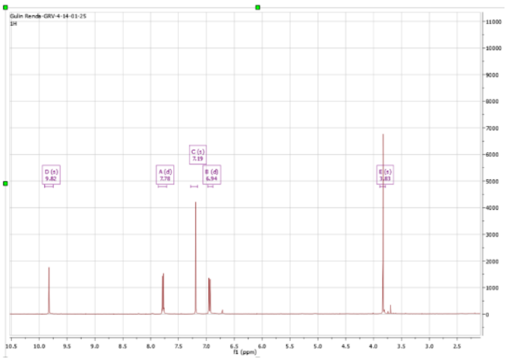

**Figure S6. $^{1}$H NMR spectrum of LO-5 (*p*-anisaldehyde).**

**KUR-2 (adenosine).** The $^{1}H$ NMR spectrum showed two characteristic singlets at $\delta_H$ 8.31 and 8.18, assigned to H-8 and H-2 of the purine moiety, respectively. The anomeric proton of the ribose unit appeared at $\delta_H$ 5.96 (d, $J$ = 6.2 Hz), while the remaining ribose proton resonances were observed between $\delta_H$ 4.74 and 3.75 ppm. The $^{13}C$-APT spectrum showed characteristic resonances of the purine moiety at $\delta_C$ 156.2, 152.1, 141.9, 136.6, and 119.7, together with five ribose carbon signals at $\delta_C$ 89.9, 86.8, 74.1, 71.3, and 62.1. In the COSY spectrum, sequential correlations from H-1'/H-2' through H-4'/H-5' supported the ribofuranosyl spin system. The spectroscopic data were consistent with those previously reported for adenosine (Ciuffreda et al., 2007) (Figure S7).

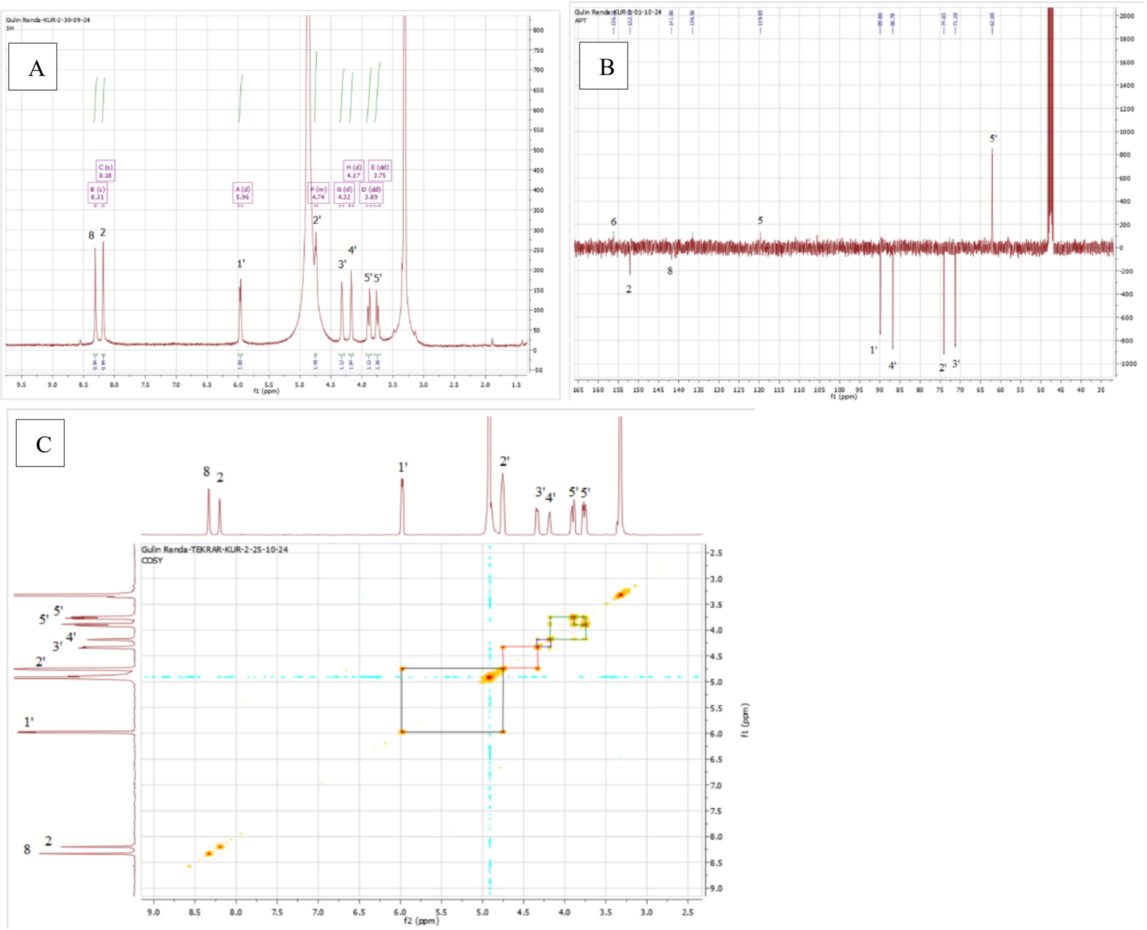


**Figure S7. NMR spectra of KUR-2 (adenosine).** (A) $^{1}H$ NMR spectrum; (B) $^{13}C$-APT spectrum; (C) COSY spectrum.

**KUR-3 (ajugol).** The $^{1}H$ NMR spectrum displayed characteristic signals of an iridoid glycoside, including the coupled olefinic proton resonances at $\delta_H$ 6.13 (dd, $J$ = 6.3, 2.0 Hz, H-3) and 4.83 (dd, $J$ = 6.3, 3.0 Hz, H-4), together with the aglycone H-1 signal at $\delta_H$ 5.43 (d, $J$ = 2.2 Hz). The anomeric proton of the glucopyranosyl unit resonated at $\delta_H$ 4.61 (d, $J$ = 7.9 Hz), supporting its β-configuration. The $^{13}C$-APT spectrum showed characteristic aglycone resonances at $\delta_C$ 139.0 (C-3), 104.5 (C-4), 92.3 (C-1), and 78.0 (C-8), together with an anomeric carbon signal at $\delta_C$ 98.0 and the remaining glucopyranosyl carbon resonances between $\delta_C$ 76.6 and 61.5. The COSY spectrum showed correlations within the aglycone spin system, including H-3/H-4, H-4/H-5, and H-5/H-6, as well as sequential correlations within the glucopyranosyl

unit. The spectroscopic data were consistent with those previously reported for ajugol (Tatlı et al., 2004b) (Figure S8).

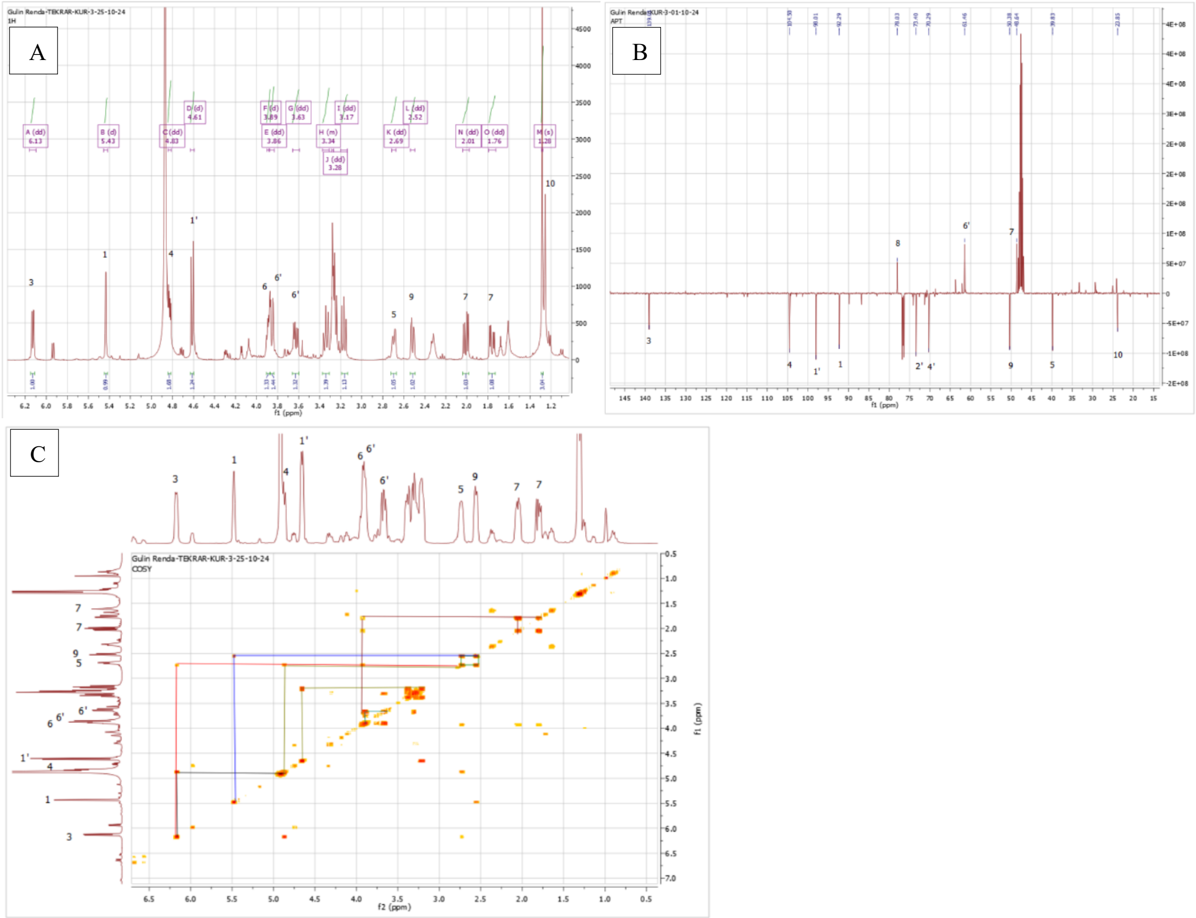


**Figure S8. NMR spectra of KUR-3 (ajugol).** (A) $^{1}$H NMR spectrum; (B) $^{13}$C-APT spectrum; (C) COSY spectrum.

**KUR-4 (aucubin).** The $^{1}$H NMR spectrum displayed characteristic signals of an iridoid glycoside, including coupled olefinic proton resonances at $\delta_H$ 6.28 (dd, $J$ = 6.1, 1.9 Hz, H-3) and 5.06 (dd, $J$ = 6.1, 3.9 Hz, H-4), together with an additional olefinic proton at $\delta_H$ 5.73 (d, $J$ = 1.4 Hz, H-7). The aglycone H-1 signal appeared at $\delta_H$ 4.92 (d, $J$ = 7.2 Hz), while the anomeric proton of the glucopyranosyl unit resonated at $\delta_H$ 4.64 (d, $J$ = 7.9 Hz), supporting its β-configuration. The $^{13}$C-APT spectrum showed characteristic aglycone resonances at $\delta_C$ 140.3 (C-3), 104.3 (C-4), 128.8 (C-7), 146.6 (C-8), and 96.3 (C-1), together with the hydroxymethylene carbon at $\delta_C$ 60.0 (C-10). The glucopyranosyl unit was supported by the anomeric carbon resonance at $\delta_C$ 98.5 and the remaining sugar carbon signals between $\delta_C$ 76.9 and 61.4. The COSY spectrum showed correlations within the aglycone spin system, including H-3/H-4, H-4/H-5, H-5/H-6, and H-5/H-9, as well as sequential correlations within the glucopyranosyl unit. The spectroscopic data were consistent with those previously reported for aucubin (Genç et al., 2010) (Figure S9).

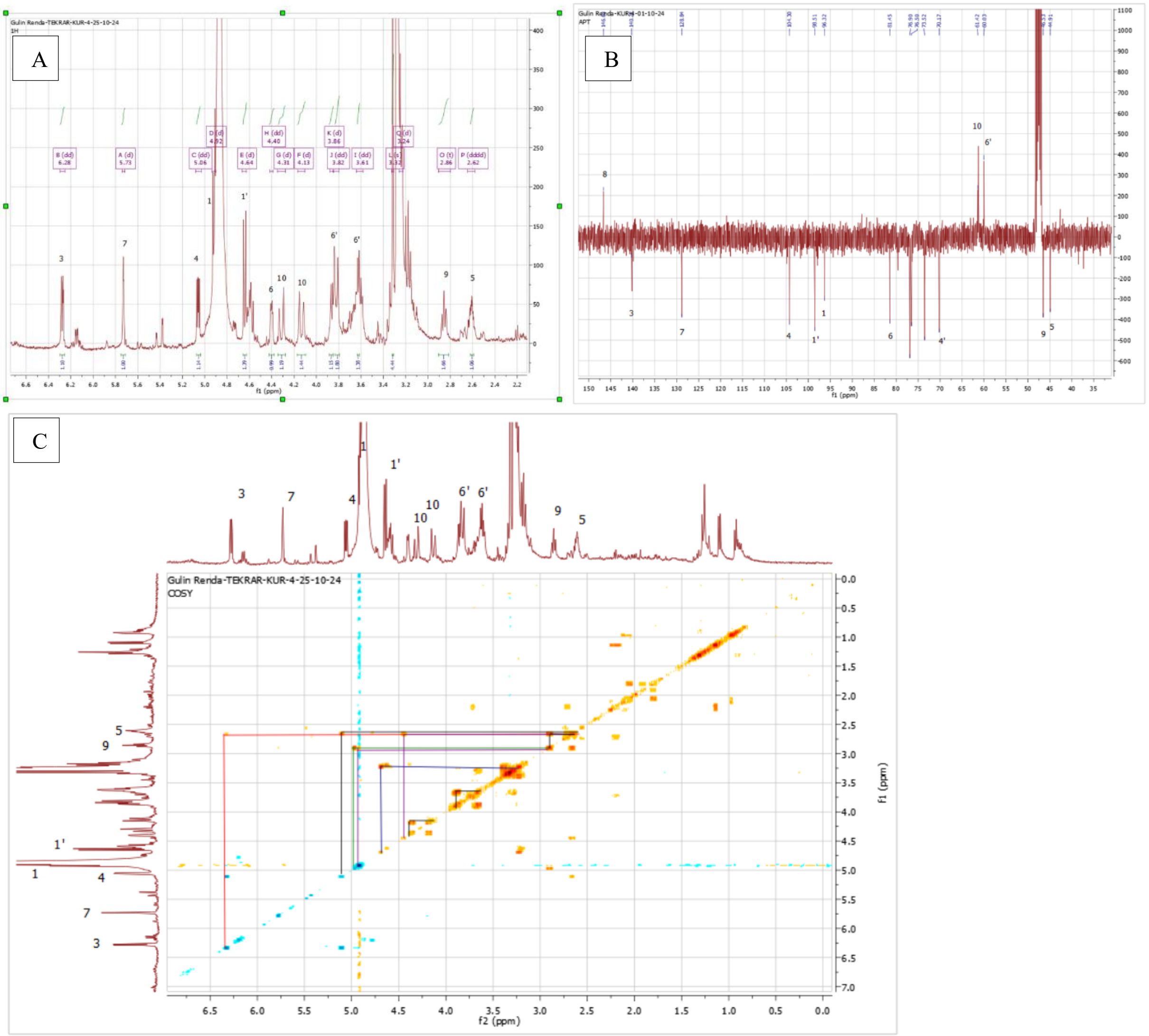


**Figure S9. NMR spectra of KUR-4 (aucubin).** (A) $^{1}$H NMR spectrum; (B) $^{13}$C-APT spectrum; (C) COSY spectrum.

**KUR-5 (ilwensisaponin C).** The $^{1}$H NMR spectrum exhibited characteristic features of a highly glycosylated triterpenoid saponin, including multiple anomeric proton resonances at $\delta_H$ 5.37, 4.90, 4.87, 4.66, and 4.53 ppm and numerous oxygenated proton signals in the $\delta_H$ 3.0-4.0 ppm region. The doublets observed at $\delta_H$ 1.29 and 1.28 ppm were consistent with methyl groups of deoxy sugar units, while several tertiary methyl singlets between $\delta_H$ 1.12 and 0.77 ppm supported an oleanane-type triterpenoid aglycone. The $^{13}$C-APT spectrum showed characteristic olefinic carbon resonances at $\delta_C$ 149.5 and 121.4, together with multiple anomeric and oxygenated sugar carbon signals, consistent with a highly glycosylated triterpenoid structure. COSY and HSQC experiments supported the individual sugar spin systems and the assignments of protonated carbons, whereas long-range correlations observed in the HMBC spectrum provided further evidence for the glycosidic linkages and connectivity between the sugar residues and the triterpenoid aglycone. Comparison of the 1D and 2D NMR data with those previously reported for ilwensisaponin C supported the identification of KUR-5 as ilwensisaponin C (Tatlı et al., 2004b) (Figure S10).

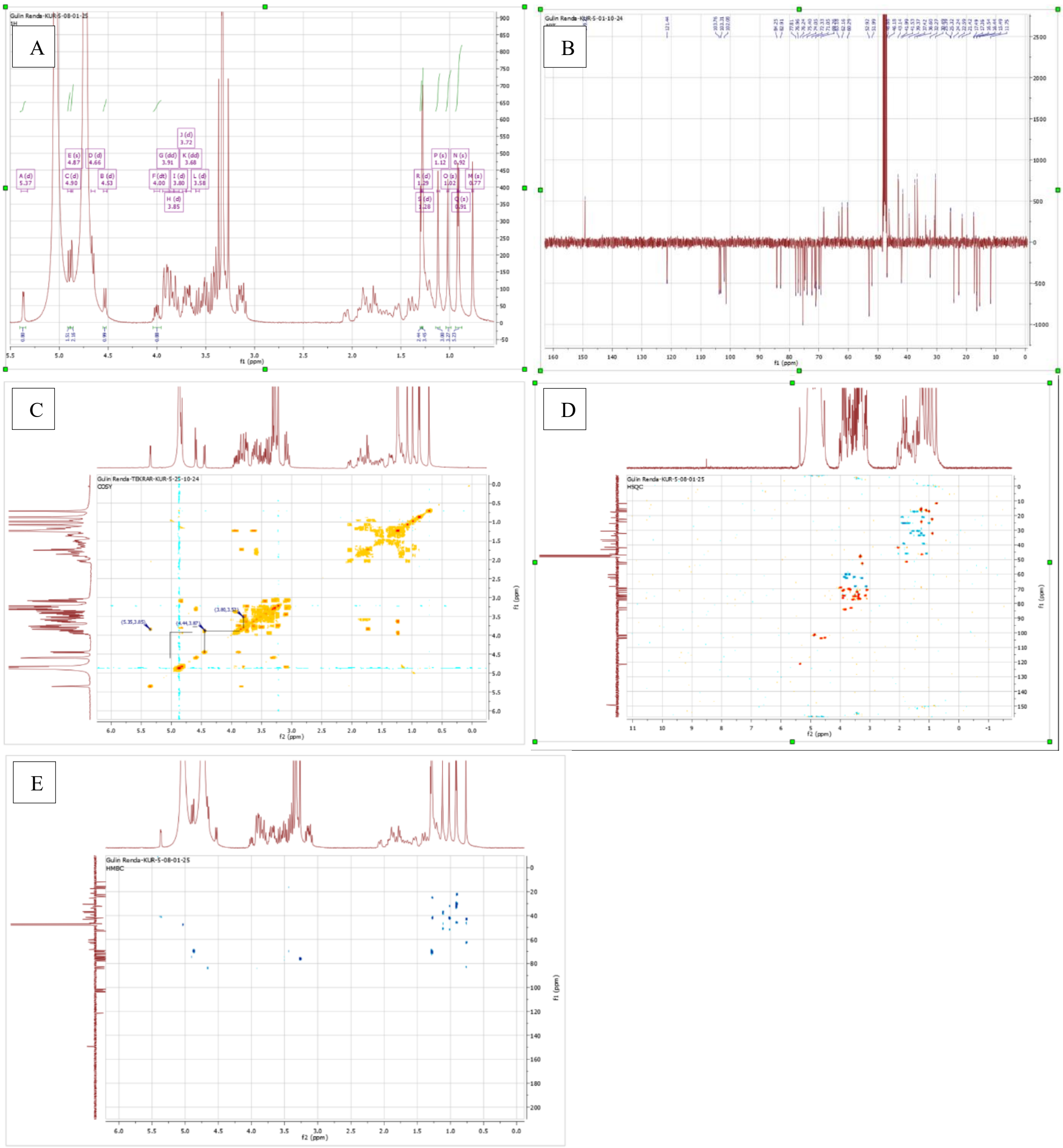


**Figure S10. NMR spectra of KUR-5 (ilwensisaponin C).** (A) $^{1}$H NMR spectrum; (B) $^{13}$C-APT spectrum; (C) COSY spectrum; (D) HSQC spectrum; (E) HMBC spectrum.

**KUR-6 (songarosaponin E).** The $^{1}$H NMR spectrum exhibited characteristic features of a highly glycosylated triterpenoid saponin, with several downfield and oxygenated proton resonances, including signals at $\delta_H$ 6.44, 5.62, and 4.80-4.55 ppm, together with numerous overlapping signals in the $\delta_H$ 3.0-4.2 ppm region attributable to sugar moieties. Multiple methyl and aliphatic proton resonances in the $\delta_H$ 0.6-1.5 ppm region supported the presence of a triterpenoid aglycone. The $^{13}$C-APT spectrum showed olefinic carbon resonances at $\delta_C$ 147.8, 139.2, 136.4, 132.2, and 121.3, together with anomeric carbon signals at $\delta_C$ 104.4, 102.9, and 102.3 and numerous oxygenated carbon resonances in the $\delta_C$ 60.7-84.1 ppm region. COSY correlations within the $\delta_H$ 3.0-4.0 ppm region supported the presence of sugar spin systems, while the HSQC spectrum confirmed direct correlations between the oxygenated proton

resonances and their corresponding carbons in the $\delta_C$ 60-105 ppm region. Taken together, the 1D and 2D NMR data were consistent with a highly glycosylated triterpenoid saponin and, by comparison with previously reported spectroscopic data, supported the identification of KUR-6 as songarosaponin E (Seifert et al., 1991) (Figure S11).

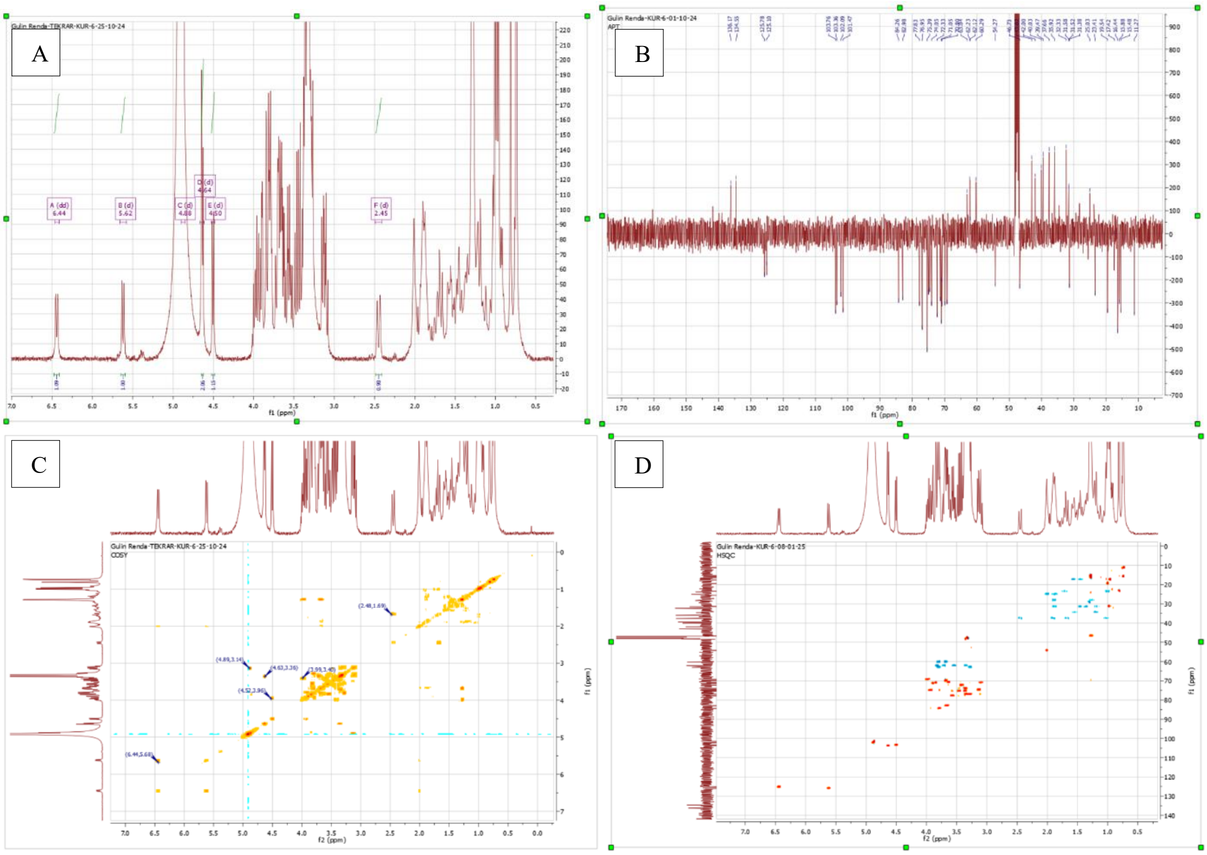

**Figure S11. NMR spectra of KUR-6 (songarosaponin E).** (A) $^1$H NMR spectrum; (B) $^{13}$C-APT spectrum; (C) COSY spectrum; (D) HSQC spectrum.

**KUR-7 (ilwensisaponin A).** The $^1$H NMR spectrum showed characteristic features of a highly oxygenated triterpenoid saponin, including coupled olefinic proton resonances at $\delta_H$ 5.91 (d, $J$ = 10.5 Hz, H-11) and 5.30 (dd, $J$ = 10.3, 2.9 Hz, H-12). Multiple anomeric proton signals at δ_H 4.44, 4.59, and 4.83 ppm, with coupling constants of approximately 7.8-7.9 Hz, supported the presence of β-configured sugar units. The $^{13}$C-APT spectrum showed olefinic carbon resonances at $\delta_C$ 132.6 (C-11) and 130.1 (C-12), together with oxygenated aglycone carbons at $\delta_C$ 82.9 (C-3), 85.5 (C-13), 76.5 (C-28), and 62.9 (C-23). COSY correlation between H-11 and H-12 supported the olefinic spin system, while multiple correlations in the sugar region were consistent with the glycosidic moieties. In the HMBC spectrum, the correlation of H-1' ($\delta_H$ 4.44) with C-3 ($\delta_C$ 82.9) supported attachment of the sugar moiety at C-3 of the aglycone. Additional long-range correlations, including H-1''/C-3' and H-1'''/C-4'', supported the interglycosidic connectivity of the sugar chain. Comparison of the 1D and 2D NMR data with those previously reported supported the identification of KUR-7 as ilwensisaponin A (Tatlı et al., 2004b) (Figure S12).

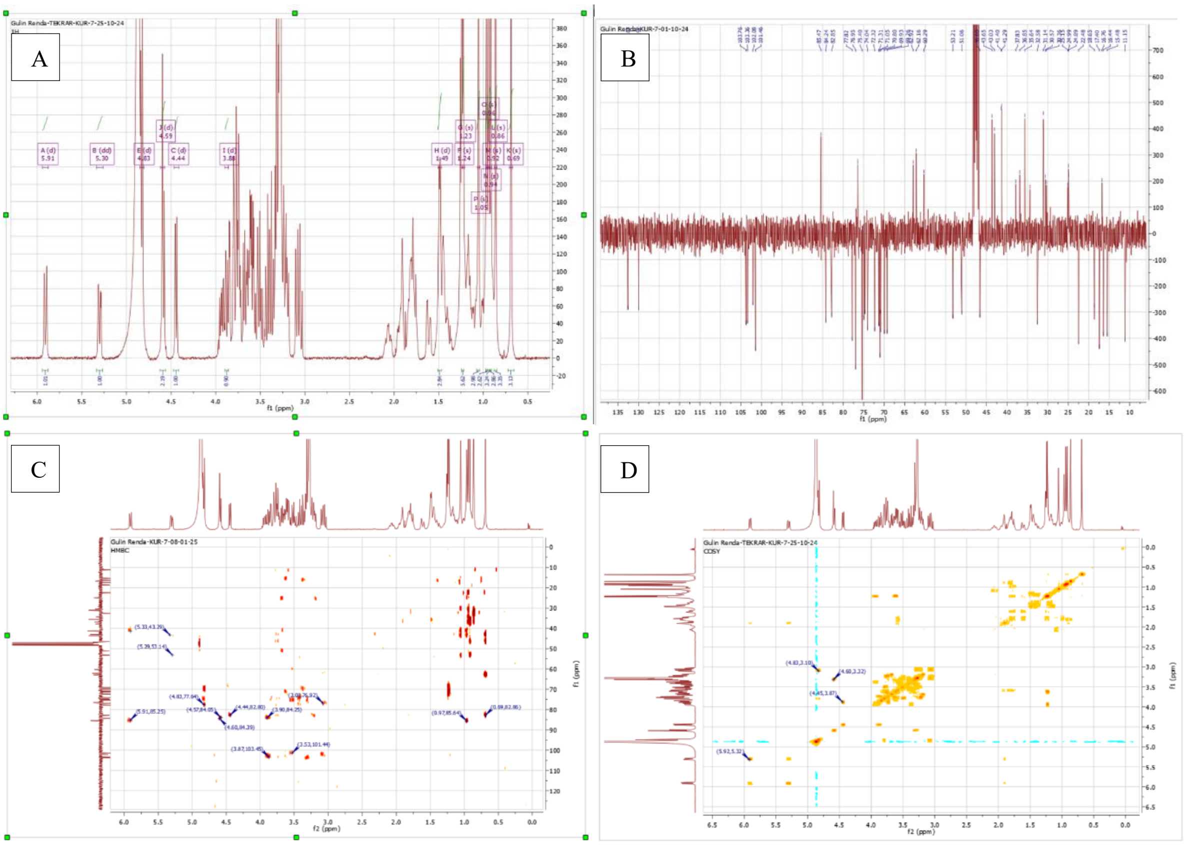

**Figure S12. NMR spectra of KUR-7 (ilwensisaponin A).** (A) $^{1}$H NMR spectrum; (B) $^{13}$C-APT spectrum; (C) HMBC spectrum; (D) COSY spectrum.

**KUR-8 (verbascoside).** The $^{1}$H NMR spectrum showed characteristic signals of a phenylethanoid glycoside, including two trans-olefinic proton resonances at $\delta_H$ 7.67 (d, $J$ = 16.0 Hz) and 6.38 (d, $J$ = 15.7 Hz), supporting the presence of a trans-caffeoyl moiety. Multiple aromatic proton signals between $\delta_H$ 7.21 and 6.70 ppm were consistent with the caffeoyl and hydroxyphenylethyl units. The anomeric proton signal at $\delta_H$ 4.38 (d, $J$ = 8.3 Hz) supported the presence of a β-configured glucopyranosyl unit, while the methyl doublet at $\delta_H$ 1.10 ($J$ = 5.7 Hz) was consistent with a rhamnosyl moiety. The $^{13}$C-APT spectrum showed resonances attributable to the carbonyl, aromatic/olefinic, and oxygenated sugar carbons expected for a caffeoyl-containing phenylethanoid glycoside. In the COSY spectrum, the correlation between the trans-olefinic protons supported the caffeoyl spin system, together with correlations within the aromatic and sugar proton systems. Comparison of the spectroscopic data with previously reported values supported the identification of KUR-8 as verbascoside (Akdemir et al., 2004) (Figure S13).

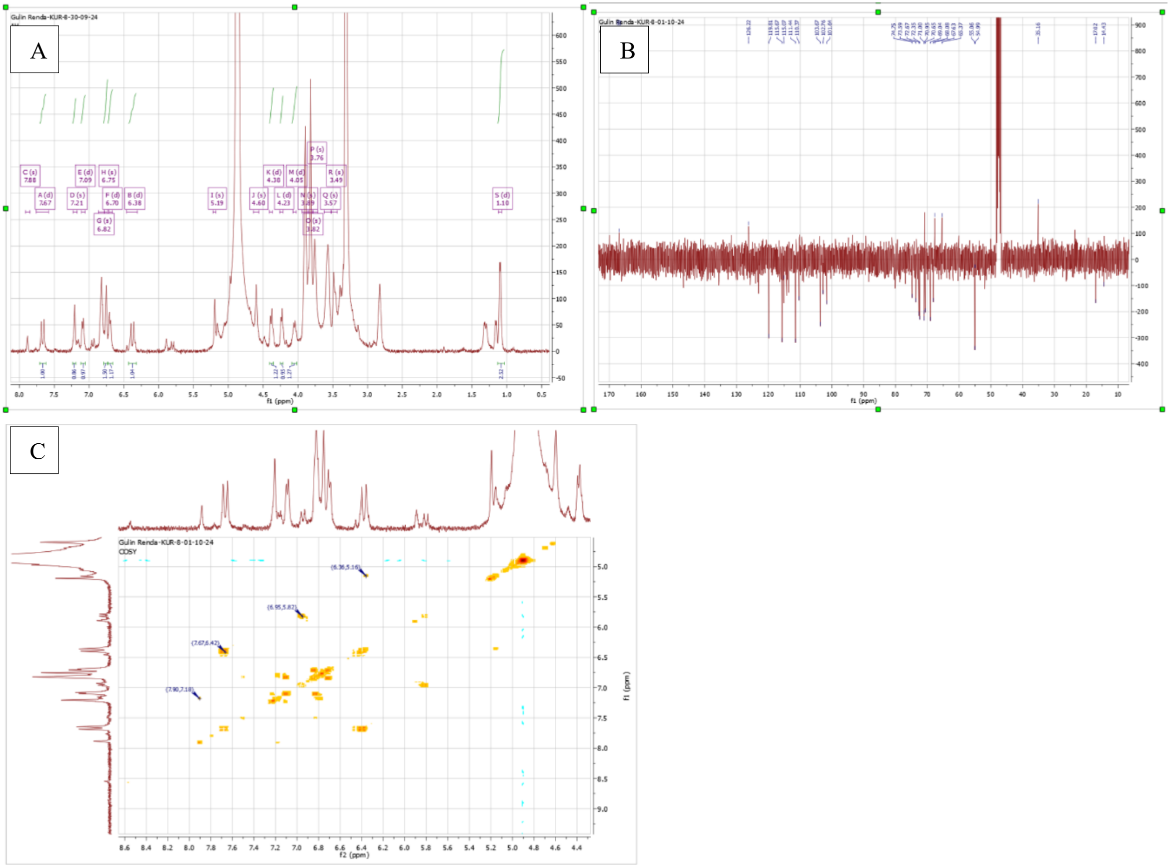


**Figure S13. NMR spectra of KUR-8 (verbascoside).** (A) $^{1}$H NMR spectrum; (B) $^{13}$C-APT spectrum; (C) COSY spectrum.

**KUR-9 (harpagoside).** The $^{1}$H NMR spectrum exhibited characteristic signals of an acylated iridoid glycoside, including two trans-olefinic proton resonances at $\delta_H$ 7.69 (d, $J$ = 16.1 Hz) and 6.54 (d, $J$ = 16.0 Hz), together with aromatic proton signals in the $\delta_H$ 7.42-7.63 ppm region, supporting the presence of an aromatic acyl moiety. Characteristic resonances of the iridoid aglycone included the olefinic H-3 signal at $\delta_H$ 6.44 and the H-1 signal at $\delta_H$ 6.20. Signals in the $\delta_H$ 3.41–4.35 ppm region were consistent with the oxygenated protons of the glucopyranosyl unit. The carbon data further supported the structure, showing an ester carbonyl resonance at $\delta_C$ 165.9, olefinic and aromatic carbon signals attributable to the acyl moiety, and an anomeric carbon resonance at $\delta_C$ 97.2 together with the remaining glucopyranosyl carbons. The COSY spectrum supported the trans-olefinic spin system of the acyl moiety and showed correlations consistent with the iridoid aglycone and sugar proton systems. Comparison of the spectroscopic data with previously reported values supported the identification of KUR-9 as harpagoside (Akdemir et al., 2004) (Figure S14).

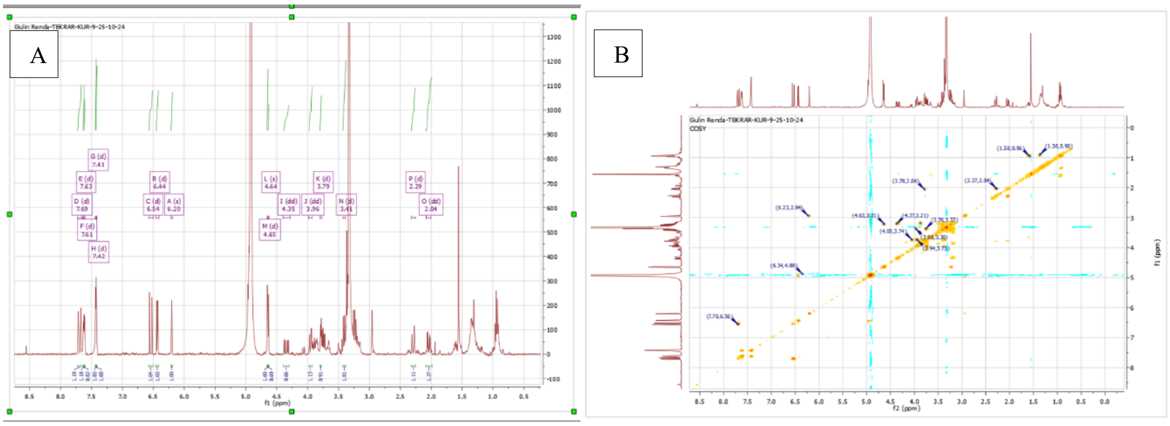


**Figure S14. NMR spectra of KUR-9 (harpagoside).** (A) $^{1}H$ NMR spectrum; (B) COSY spectrum.

## HPLC-DAD Assessment of Chromatographic Purity

**Chromatographic conditions.** Chromatographic analyses were performed on a Shimadzu Prominence-20 series HPLC system (Shimadzu, Kyoto, Japan) equipped with an SPD-M20A diode-array detector (DAD). Separation was achieved on an Inertsil ODS-3 column (150 × 4.6 mm, 5 µm; GL Sciences, Tokyo, Japan) at 25 °C, with a flow rate of 1.0 mL $min^{-1}$ and an injection volume of 20 µL. The mobile phase consisted of 20 mM phosphate buffer (pH 2.5) (solvent A) and acetonitrile (solvent B). Gradient elution was applied, with solvent B increased linearly from 10% to 100% over 0-30 min. Detection was performed at 254 nm. Between runs, the column was re-equilibrated under the initial conditions (10% B) for 10 min. Chromatographic purity was estimated by peak-area normalization at 254 nm.

**Table S2. HPLC-DAD chromatographic purity of selected isolated compounds determined by peak-area normalization at 254 nm.**

| Compound | Code | Chromatographic purity (%) |
|---|---|---|
| Luteolin 7-O-β-D-glucopyranoside | LO-1 | 93.76 |
| 1-O-Caffeoyl-β-D-glucopyranose | LO-4 | 87.11 |
| *p*-Anisaldehyde | LO-5 | 91.92 |
| Ajugol | KUR-3 | 86.25 |

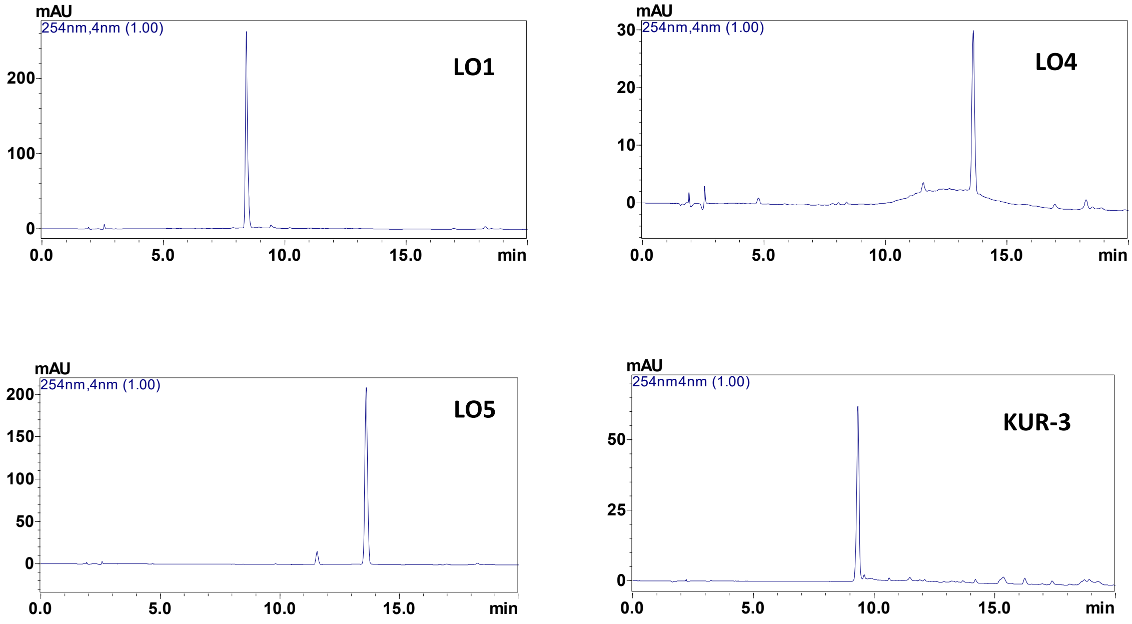


**Figure S15. HPLC-DAD chromatograms of selected isolated compounds used for chromatographic purity assessment.** LO-1, luteolin 7-O-β-D-glucopyranoside (93.76%); LO-4, 1-O-caffeoyl-β-D-glucopyranose (87.11%); LO-5, *p*-anisaldehyde (91.92%); and KUR-3, ajugol (86.25%). Chromatograms were recorded at 254 nm, and chromatographic purity was estimated by peak-area normalization.

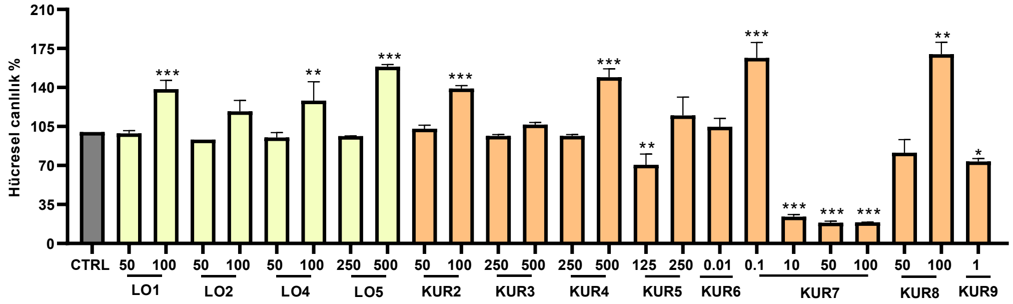


**Figure S16. Cytotoxicity profiles of the isolated compounds in BEAS-2B cells.** Cells were exposed to the indicated concentrations of the isolated compounds for 24 h, and cell viability was determined using the MTT assay. Data are presented relative to the vehicle control (0.5% DMSO). Statistical significance versus the control group is indicated as $p < 0.05$, $p < 0.01$, and $p < 0.001$.

**Table S3. Concentrations of the isolated compounds selected for cytoprotective screening in BEAS-2B cells.**

| Compound code | Identified compound | Selected concentration (µM) |
|---|---|---|
| LO1 | Luteolin 7-*O*-β-D-glucopyranoside | 100 |
| LO2 | Ethyl 2,4-dihydroxybutanoate | 100 |
| LO4 | 1-*O*-Caffeoyl-β-D-glucopyranose | 100 |
| LO5 | *p*-Anisaldehyde | 500 |
| KUR2 | Adenosine | 100 |
| KUR3 | Ajugol | 500 |
| KUR4 | Aucubin | 500 |
| KUR5 | Ilwensisaponin C | 250 |
| KUR6 | Songarosaponin E | 0.01 |
| KUR7 | Ilwensisaponin A | 0.1 |
| KUR8 | Verbascoside | 100 |
| KUR9 | Harpagoside | 1 |
